# Ultrafast and Nanoscale Energy Transduction Mechanisms and Coupled Thermal Transport across Interfaces

**DOI:** 10.1021/acsnano.3c02417

**Published:** 2023-07-17

**Authors:** Ashutosh Giri, Scott G. Walton, John Tomko, Niraj Bhatt, Michael J. Johnson, David R. Boris, Guanyu Lu, Joshua D. Caldwell, Oleg V. Prezhdo, Patrick E. Hopkins

**Affiliations:** † Department of Mechanical, Industrial and Systems Engineering, University of Rhode Island, Kingston, Rhode Island 02881, United States; ‡ Plasma Physics Division, Naval Research Laboratory, Washington, DC 22032, United States; § Department of Mechanical and Aerospace Engineering, University of Virginia, Charlottesville, Virginia 22904, United States; ⊥ Department of Mechanical Engineering, Vanderbilt University, Nashville, Tennessee 37235, United States; # Interdisciplinary Materials Science, Vanderbilt University, Nashville, Tennessee 37235, United States; ∇ Vanderbilt Institute of Nanoscale Science and Engineering, Vanderbilt University, Nashville, Tennessee 37235, United States; @ Department of Chemistry, University of Southern California, Los Angeles, California 90089, United States; % Department of Physics and Astronomy, University of Southern California, Los Angeles, California 90089, United States; $ Department of Materials Science and Engineering, University of Virginia, Charlottesville, Virginia 22904, United States; & Department of Physics, University of Virginia, Charlottesville, Virginia 22904, United States

**Keywords:** interfacial heat transfer, energy transduction, coupled local equilibrium, electron−phonon coupling, plasmon polaritons, ballistic thermal injection, plasmas, *ab initio* electron−vibrational dynamics at interfaces, solid−gas interactions

## Abstract

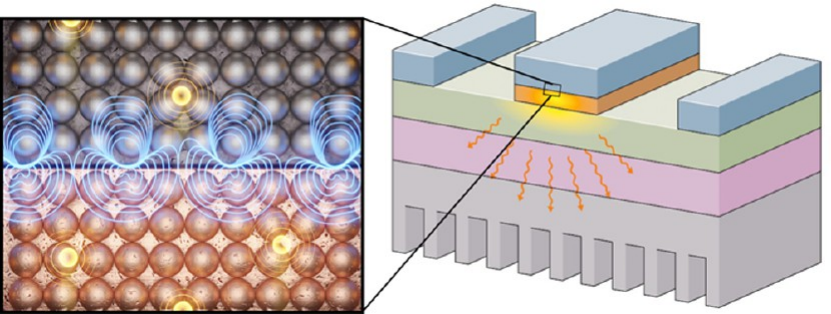

The coupled interactions among the fundamental carriers of charge, heat, and electromagnetic fields at interfaces and boundaries give rise to energetic processes that enable a wide array of technologies. The energy transduction among these coupled carriers results in thermal dissipation at these surfaces, often quantified by the thermal boundary resistance, thus driving the functionalities of the modern nanotechnologies that are continuing to provide transformational benefits in computing, communication, health care, clean energy, power recycling, sensing, and manufacturing, to name a few. It is the purpose of this Review to summarize recent works that have been reported on ultrafast and nanoscale energy transduction and heat transfer mechanisms across interfaces when different thermal carriers couple near or across interfaces. We review coupled heat transfer mechanisms at interfaces of solids, liquids, gasses, and plasmas that drive the resulting interfacial heat transfer and temperature gradients due to energy and momentum coupling among various combinations of electrons, vibrons, photons, polaritons (plasmon polaritons and phonon polaritons), and molecules. These interfacial thermal transport processes with coupled energy carriers involve relatively recent research, and thus, several opportunities exist to further develop these nascent fields, which we comment on throughout the course of this Review.

## I Introduction

The coupled interactions between the fundamental carriers of charge, heat, and electromagnetic fields are critical processes that dictate the functionality, efficiency, and design of a wide array of material composites and devices. At interfaces and surfaces in nanomaterials, these intertwined mechanisms are the foundation of the modern technologies that are continuing to provide transformational benefits in computing, communication, health care, clean energy, power recycling, sensing, and manufacturing, to name a few. For example, transistors rely on the interaction of field with carriers across metal/oxide/active region boundaries,^1−4^ catalysis can be greatly enhanced through non-equilibrium charge injection across interfaces before thermal equilibration with phonons,^5−7^ and the ability of photoexcited charges to efficiently couple across a semiconductor/metal interface dictates the efficiency of solar energy harvesting devices.^8−14^ In these applications, the interaction and transport among the various carriers across and around the interface between two materials give rise to increased energy density, which can result in deleterious temperature rises that can impact the efficiency of the devices.^15−17^ Specifically, it is well known that the resulting thermal boundary resistances (TBRs) that occur at interfaces of two different materials or phases of matter are the limiting factor that dictate, for example, the scalability of transistors in CMOS architectures that drives the semiconductor industry’s capability to keep pace with Moore’s law;^18−20^ the ability of wide- and ultrawide-bandgap-based power devices from achieving their intrinsic material potentials in RF power converters for applications ranging from military radar systems to wireless communication for 6G and beyond;^17^ and the efficiency of photothermal therapeutics to maintain localized and controlled temperature rise to target selective treatment of cancerous cells while ensuring the healthy tissues are unperturbed.^21^

Clearly, the energy coupling among the different carriers at interfaces can be the critical heat transfer pathway, dictating the efficacy of various processes and applications. This finding of coupling between different types of energy carriers driving TBR across interfaces dates back to Kapitza’s original work in 1941 demonstrating that a temperature drop can exist at an interface between Cu and liquid helium.^22^ In this case, the temperature drop and resulting TBR were explained to be driven by acoustic waves (which are the primary carriers of heat at these cryogenic, single-digit Kelvin temperatures and below) transmitting from the solid Cu to the acoustic waves in the liquid He. This original work by Kapitza and several other works studying this low-temperature TBR effect were often focused on low-temperature heat transfer across solid/liquid or sold/gas interfaces, thus involving the transduction of energy from solid acoustic waves (dispersionless phonons) to pressure waves in different phases of matter.^23−30^ The ensuing theories from these studies resulted in translatable models to describe phonon transmission across solid/solid interfaces due to the similarities in acoustic wave reflection and transmission interactions at interfaces.^31^ From this, classic theories that are often used to predict the phonon thermal boundary conductance across solid/solid interfaces were born, such as the acoustic mismatch model (AMM),^24,32,33^ the diffuse mismatch model (DMM),^31^ and the phonon radiation limit (PRL).^25^ These models have been routinely applied to describe TBR across solid/solid interfaces.^34−40^ However, to capture the true dynamics of heat flow across these interfaces, several re-derivations of these theories have been proposed to account for more complex interface features and phonon scattering events.^41−49^

The limitations of this historical understanding of heat flow across interfaces (which ultimately dates back to Kapitza’s original concepts), however, prevent a greater foundational and nanoscopic understanding of TBR across interfaces when different energy carriers are coupled near and across interfaces. For example, the creation of hybridized vibrational states around interfaces (often referred to as “interfacial vibrational modes”) is not accounted for in continuum-based mismatch models, and a more accurate understanding of how these modes influence TBR must be approached with atomistic models and experimental probes, which we review in Section II. Similarly, the coupled interactions between electrons and phonons can impact TBR, a process that has been theorized to be strongly dependent on electron–phonon scattering either near or across interfaces,^50−52^ as we discuss in Section III. Coupled carriers of heat can also be driven by extrinsic stimuli, such as polaritonic coupling of electromagnetic fields with electrons (plasmon polaritons)^53^ or phonons (phonon polaritons),^54−56^ which offer a potential interfacial process that could drive TBR, a currently bourgeoning area of research we overview in Section IV. While the examples above focus mainly on the breakdown of the historical understanding and theories of TBR across solid/solid interfaces when thermal carriers are coupled at or near interfaces, when approaching interfaces between two different phases of matter (solid/gas and solid/liquid) at non-cryogenic temperatures, the nanoscale interactions at interfaces must be accounted for. Thus, the concepts of acoustic wave transmission across these solid/gas and solid/liquid interfaces originally applied to explain century old liquid helium data must be modified. Notably, nanoscale interactions that can be modified from surface chemistry, pressure, and phase changes in the liquid and gas must be considered to properly account for heat transfer, which we overview in Section V. We end Section V by describing recent studies on the energy transfer at solid surfaces exposed to plasma,^57−59^ where this fourth phase of matter delivers a plethora of energy carriers to the solid and can give rise to different energy transfer processes as a function of time during plasma exposure, including the recent demonstration of “plasma cooling”, where a directed plasma jet can be used to transiently cool a surface.

**Figure 1:**
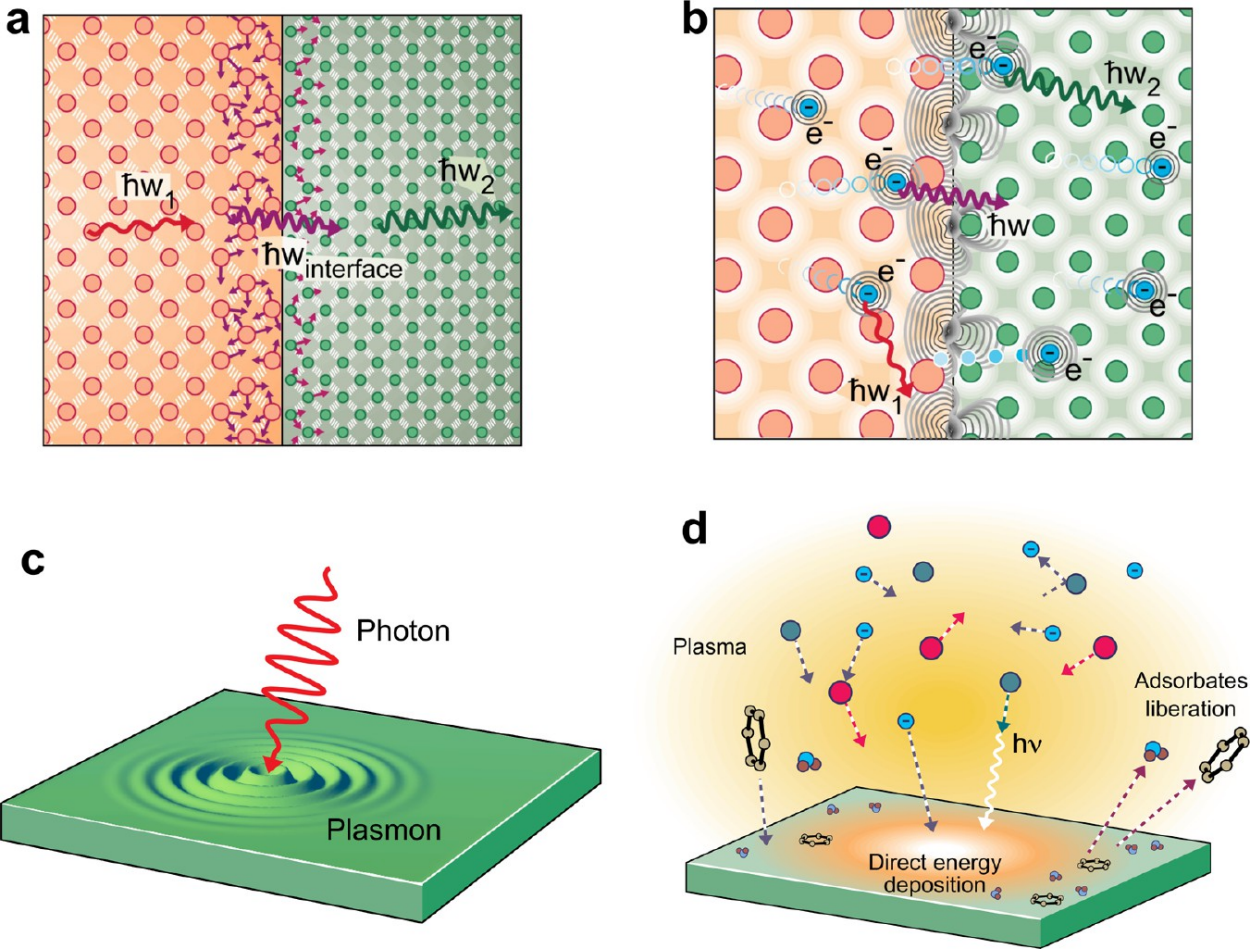
This article reviews recent works that have focused on understanding energy transfer processes across material interfaces resulting from (a) hybrid interfacial phonon modes, (b) coupled electron–phonon interactions, (c) polaritonic coupling of electromagnetic fields with electrons or phonons (plasmon polaritons or phonon polaritons, respectively), and (d) plasma–surface interactions.

It is the purpose of this Review to describe recent works that have reported on the ultrafast energy transduction mechanisms across interfaces when different thermal carriers couple near or across interfaces to drive the resulting TBR. Notably, as suggested in the paragraph above, many of these interfacial thermal transport processes with coupled energy carriers involve relatively recent research, and thus, several opportunities exist to further develop these nascent fields, which we comment on throughout the course of this Review. Our article is separated into four main sections focusing on different coupled thermal transport processes across interfaces, as depicted in Figure 1. It is not the purpose of this article to review the current knowledge of TBR across interfaces, as several extensive works currently exist that review historical advances and more recent nanoscale heat transfer advances in this space.^31,41,42,60−62^ However, the topic of ultrafast energy transduction and coupled thermal carriers driving TBR has not been a major focus of any recent perspective or review, most likely due to the relative infancy of many of these results reviewed herein. Thus, this manuscript serves to present the community with our perspective and perceived opportunities for further developing the field of ultrafast energy transduction mechanisms and coupled thermal transport across interfaces.

## II Hybrid Phonon Modes and Interfacial Vibrations

Interfaces in heterostructures can give rise to hybridized vibrational states that result from changes in the atomic scale symmetry and are otherwise absent from the vibrational spectrum of either “bulk” material. The existence of these interfacial modes that are ascribed to the chemical and structural makeup of the interface has been demonstrated through various experimental and computational approaches with ample evidence of their global response, such as influencing the magnetic and ferroelectric properties,^63,64^ controlling the metal–insulator transition,^65^ strengthening the superconducting nature,^66^ and manipulating the overall heat conduction in various heterostructures.^67^ However, for the purposes of this Review, we will only focus on hybrid vibrational states that couple across heterostructures and interfaces with broken symmetries and their influence on the overall heat transfer mechanisms. In particular, we highlight the recent developments in characterization and computational tools that have led to the realization of hybrid vibrational states that significantly influence interfacial thermal transport, thus questioning the applicability of the often used phonon gas models that can only describe propagating modes in solids and the transmission of these modes across solid/solid interfaces. Furthermore, we will also focus on nanophononic metamaterials in which strategically placing nanoresonators on the surface of thin films or nanowires can lead to the coupling of low-frequency hybridized modes and phonon resonances across the boundaries, which are otherwise unaffected by conventional nanostructuring strategies.

### II.a Localized Interface Vibrational Modes as Efficient Energy Exchange Channels

The characterization of vibrational states has generally relied on infrared and Raman spectroscopies,^71,72^ inelastic X-ray and neutron scattering,^73,74^ and electron tunneling experiments with spatial resolutions that are typically on the order of several micrometers.^75^ Due to the lack of spatial resolution, it has proven difficult to identify and visualize localized vibrational states at interfaces in heterostructures, which requires very high spatial resolutions (on the nanometer length scales) capable of revealing the dynamics of individual atoms. This capability was achieved in 2014 from the breakthrough work of Krivanek et al.^76^ where they demonstrated that vibrational spectroscopy could be combined with sub-nanometer spatial resolution via spatially resolved electron energy loss spectroscopy (EELS), thus allowing the measurement of local phonon spectra at the atomic scale. More recently, utilizing this technique, the local vibrational spectra at a high-quality epitaxial Si/Ge interface^68^ and the interface phonon dispersion relation for the cubic boron nitride/diamond heterointerface^77^ with features that appear ∼1 nm around the interface have been studied. These breakthroughs have significantly contributed to our understanding of localized and hybrid phonon states that are uncharacteristic of either “bulk” constituent, thus offering insight into the lattice dynamics of heterostructures. Moreover, these findings can also potentially impact applications such as thermal management in electronics and topological phononics.

**Figure 2:**
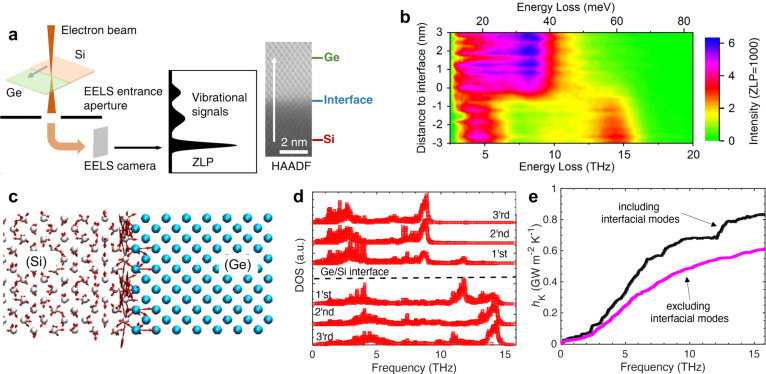
(a) Schematic diagram of EELS measurements for the high-quality epitaxial Si/Ge interface for obtaining the spatially resolved local vibrational spectrum at the interface. (b) Line profile of the vibrational spectrum across the Si/Ge interface showing localized interfacial modes at 12 THz in the vicinity of the interface. Adapted under the terms of the Creative Commons CC BY license from ref (68). Published 2021 Springer Nature. (c) Eigenvectors for interfacial modes at 12.01 THz. Adapted under the terms of the Creative Commons CC BY license from ref (69). Published 2016 Springer Nature. (d) Phonon density of states calculated from atomistic simulations reveal that interfacial modes are mainly localized around the first atomic plane near the interface for Si/Ge. (e) These modes can have a substantial contribution to the total thermal boundary conductance (*h*_K_) as shown by Gordiz et al.^69^ Adapted under the terms of the Creative Commons CC BY license from ref (69). Published 2016 Springer Nature.

For the Si/Ge interface mentioned above, Cheng et al.^68^ utilized a combination of Raman spectroscopy, scanning transmission electron microscope (STEM) with a probe size of 1.5 Å, and high-energy-resolution EELS to demonstrate the existence of interfacial modes at ∼12 THz that are confined at the interface. The schematic diagram of their experiment is shown in Figure 2a where the peaks in the EELS signal are energies of the vibrational phonon modes. This is evident from Figure 2b taken from their work that shows a line-scan of vibrational spectra going from the silicon side to the germanium layer with a very high spatial resolution. The vibrational spectra as a function of distance to the Si/Ge interface shown in the figure have a clear peak at ∼12 THz indicative of the localized interfacial mode.

Prior to this work, numerous theoretical and computational studies have predicted the existence of interfacial modes that are otherwise not present in the bulk of the constituent materials comprising the interface.^69,70,78−85^ An example of such a localized mode at 12.01 THz is depicted in Figure 2c (taken from lattice dynamics calculations carried out in ref (69)), which shows the eigenvectors and their localized nature for a Si/Ge interface. These interfacial modes occur at frequencies above the maximum frequency of the softer solid (Ge) and are localized mainly around the first atomic layer of a pristine Si/Ge interface, as shown in Figure 2d, which leads to the speculation that any contribution to heat conduction across the interface is a result of anharmonic interactions. Spectral and modal decomposition of the heat flux across these interfaces carried out via atomistic simulations confirms this speculation and has shown that these modes can contribute substantially (by up to 15%) to the total interfacial conductance across the Si/Ge interface (Figure 2e). These results highlight the opportunity of engineering interfacial vibrational modes to influence the overall thermal conductivity of heterostructures with high-density material interfaces. Such a strategy has been applied recently to modify the interfacial modes in amorphous multilayers composed of alternating layers of hydrogenated amorphous silicon carbide and hydrogenated amorphous silicon oxycarbide that are widely used as low-dielectric-constant materials in high-density, highly integrated microelectronic devices.^35^

### II.b Emergent Interfacial Vibrational Modes in Superlattices

**Figure 3:**
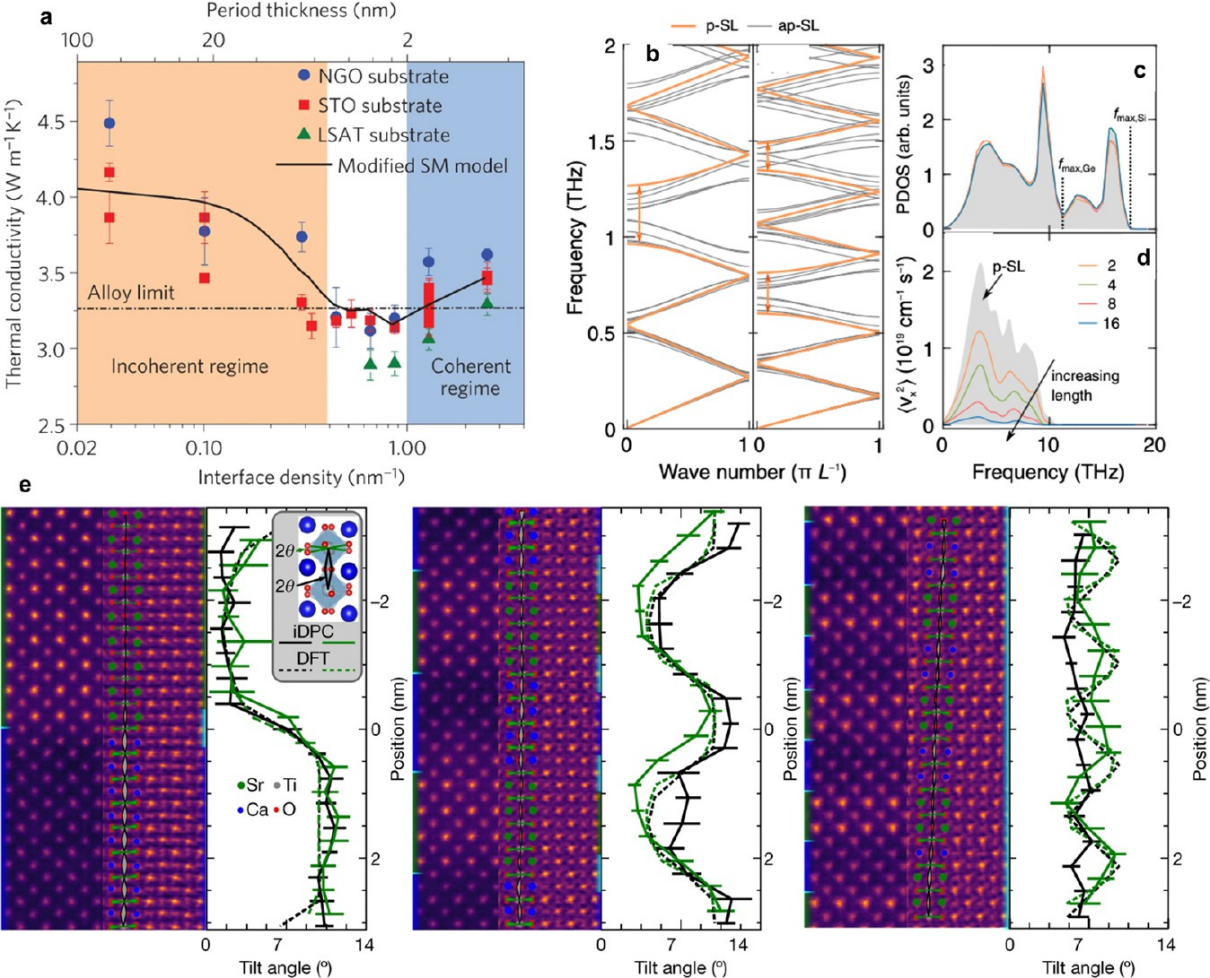
(a) Thermal conductivity as a function of interface density for oxide superlattices of calcium titanate and strontium titanate (CTO/STO) showing a minimum in thermal conductivity that can be achieved at high interface densities.^86^ Adapted with permission from ref (86). Copyright 2014 Springer Nature. (b) Phonon dispersion for periodic (thick line) and aperiodic (thinner lines) Si/Ge superlattices showing miniband formations for low-frequency longitudinal and transverse phonons.^87^ (c) Phonon density of states for the Si/Ge superlattices and (d) the calculated group velocities (taken from ref (87)). While there is no change in the phonon density of states, increasing the number of periods in the aperiodic superlattice leads to further miniband formation and drastic reductions in the phonon group velocities. This results in the quenching of thermal transport through Anderson localization of phonons. Adapted under the terms of the Creative Commons CC BY license from ref (87). Published 2019 American Physical Society. (e) Annular dark-field and integrated differential phase contrast images showing octahedral tilt angles for CTO/STO superlattices with period thicknesses of 27, 4, and 2 pseudocubic unit cells (going from left to right, respectively).^67^ While the two thicker period superlattices show sinusoidal tilt angles representative of CTO and STO layers, the tilt angle is constant at 7° for the thinnest period superlattice, where the layers lose uniqueness and the superlattice adopts the structure and vibrational response of the interface. Adapted with permission under a Creative Commons CC BY License from ref (67). Copyright 2022 Springer Nature.

Vibrational modes and their interplay with interfaces and coherent phonons also present a challenge in understanding the fundamental vibrational physics in superlattices with multiple interfaces. In this regard, phonon transport across short-period superlattices has garnered much attention over the past two decades mainly due to the observation of a crossover between particle-like (incoherent and diffusive) transport to a wave-like (coherent) transport regime that is possible through the strategic choice of the period thickness in superlattices.^86,88−90^ Such a control of the dual-wave-particle nature of phonon transport can be used for phonon engineering and controling the overall thermal conductivity of superlattices. For instance, in superlattices, the internal interfaces can diffusively scatter phonons in the classical size effect regime (or the Casimir regime), or the internal interfaces can cause interference of phonon waves, thus modifying the intrinsic phonon dispersion of the superlattice material.^89,91,92^ However, a clear experimental observation of the latter case requires pristine internal interfaces with minimal intermixing between the different material layers of the periodic superlattice. This is because interface roughness can diffusely scatter phonons and lead to the reduction in the experimentally measured thermal conductivities in superlattices that are lower as compared to their bulk parent materials.^93−96^ In this regard, measurements of thermal conductivity on epitaxially grown calcium titanate/strontium titanate (CTO/STO) superlattices (Figure 3a taken from ref (86)) with pristine interfaces have shown that a minimum in thermal conductivity is achieved at high interface densities. For thicker period CTO/STO superlattices, the thermal conductivity decreased with increasing interface densities, which is consistent with the particle nature of phonons, where they scatter at the internal interfaces. However, increasing the interface density beyond ∼1 nm^–1^ was shown to lead to higher thermal conductivities indicating a change in the nature of the vibrational modes of the parent materials that can dictate thermal transport in short period CTO/STO superlattices. In other words, if phonons scatter at the individual interfaces (as is evident in thick period superlattices), the transport can be well-described by diffuse (or incoherent) scattering in the classical Casimir regime that leads to a reduction in thermal conductivity as interface density is increased.^97^ In contrast, for the case when the phonon dispersion is modified by the formation of minibands in the phonon dispersion (as exemplified in Figure 3b for representative short-period Si/Ge superlattices),^87^ or new modes that could arise due to interfacial interactions, thermal transport can be dictated by wave interference effects.

As shown in Figure 3b, in the case of miniband formation in short-period superlattices, reduced group velocities of phonons results. However, the intriguing aspect about the emergence of new vibrational modes in short-period superlattices is that their thermal conductivity increases with decreasing period thickness (or increasing interface density as shown in Figure 3a for the CTO/STO superlattices). In the kinetic theory description, thermal conductivity is approximated by , where *C* is the heat capacity, *v* is the group velocity, and λ is the mean free path of vibrational modes. In this picture, thermal conductivity is expected to decrease with decreasing *v* due to the miniband formation in short-period superlattices. However, the increase in thermal conductivity with shorter periods is attributed to phonons with mean free paths that are longer than the period thickness of the superlattices, which can dictate the overall heat conduction. Therefore, this interplay between the particle and wave types of thermal transport at and across interfaces in superlattices can be utilized to minimize the thermal conductivity of superlattices well below the alloy limit (Figure 3a), which could be beneficial for thermoelectric applications.

In terms of quenching thermal transport through engineering material interfaces in the coherent regime, Anderson localization of phonons in superlattices has received considerable interest over the recent years.^87,98−100^ This phenomenon originates from the wave interference,^101^ which is mainly evident from the observation of decreasing thermal conductivity with increasing total thickness (i.e., the number of periods in superlattices).^99,102^ This has been demonstrated computationally for aperiodic Si/Ge superlattices where increasing the system size not only leads to miniband formations (and folding of the Brillouin zone as observed for periodic superlattices) but also leads to further miniband formations and larger reductions in the group velocities (Figure 3b). Although increasing the number of periods does not lead to any changes in the phonon density of states of the overall structure (Figure 3c), the massive reduction in the group velocities (with an increasing number of periods as shown for aperiodic Si/Ge superlattices in Figure 3c) ultimately quenches heat conduction. This phenomenon originates from the exponential decay of vibrational eigenmodes in the aperiodic superlattices, whereas these modes can extend throughout the entire superlattice structure in the periodic counterparts. Furthermore, machine learning has also been implemented to maximize the Anderson localization by optimizing the aperiodicity to achieve the minimum possible thermal conductivity in superlattices.^100,103^ Similarly, experiments carried out on GaAs/AlAs superlattices with embedded ErAs quantum dots with characteristic lengths similar to the individual layer thickness have also shown signatures of Anderson localization albeit at cryogenic temperatures.^102^ However, at higher temperatures, inelastic phonon–phonon scattering processes become dominant, and phonon transport is no longer in the coherent regime. Taken together, these recent studies have demonstrated that vibrational localization through engineering material interfaces provides a route to quench phonon heat conduction through understanding and manipulating their wave nature, which has so far only been commonplace for photons and electrons throughout previous decades.

In the extreme limit of vibrational localization, in very short-period superlattices, it could be expected that hybrid phonon states emerge where the vibrational response of the interface is adopted by the entire heterostructure and the individual layers lose their uniqueness. Recently, Hoglund et al.^67^ have demonstrated this extreme phonon transport in CTO/STO superlattices through a combination of advanced STEM imaging and EELS, ultrafast pump–probe spectroscopy, and density functional theory (DFT) calculations. With these techniques, they provide direct visualization of emergent interfacial vibrational modes in the oxide superlattices through changes in the atomic scale symmetry of superlattices with period thicknesses comparable to those of the structurally diffuse interfaces. The observed octahedral coupling occurs at the individual layers and is uncharacteristic of either “bulk” constituent (Figure 3e). In other words, through high spatial and spectral resolutions, they demonstrate that the heterostructures can adopt the vibrational response of the interface through the STO layers inheriting tilts from the CTO layers. More specifically, they demonstrate this through annular dark-field and integrated differential phase contrast from segmented STEM detectors that can image both light and heavy elements, providing knowledge of the local symmetry, which dictates the vibrational states. The images (as shown in Figure 3e for 27, 4, and 2 unit cells of CTO/STO superlattices) quantify the octahedral tilts where the octahedral tilts transform from a sinusoidal tilt profile (for the thicker 27 and 4 unit cell structures) to a near constant tilt angle of 7° for the thinnest period superlattice that extends throughout the entire structure. This incorporation of atomic displacements in STO layers through the interface-mediated influence from the CTO layers results in the predominance of interfacial vibrations that ultimately determine the overall heat conduction in these superlattices, where a clear crossover from incoherent to coherent phonon transport is observed as the period thickness decreases (Figure 3a). As mentioned above, the coherent regime had been previously attributed mainly to zone-folding of phonon dispersion, leading to an increased group velocity. Hoglund et al.,^67^ however, provide direct evidence connecting such intrinsic phononic processes to vibrational modes and structural changes in superlattices, which had been previously missing from the literature.

### II.c Hybridized Phonon Modes in Nanophononic Metamaterials

**Figure 4:**
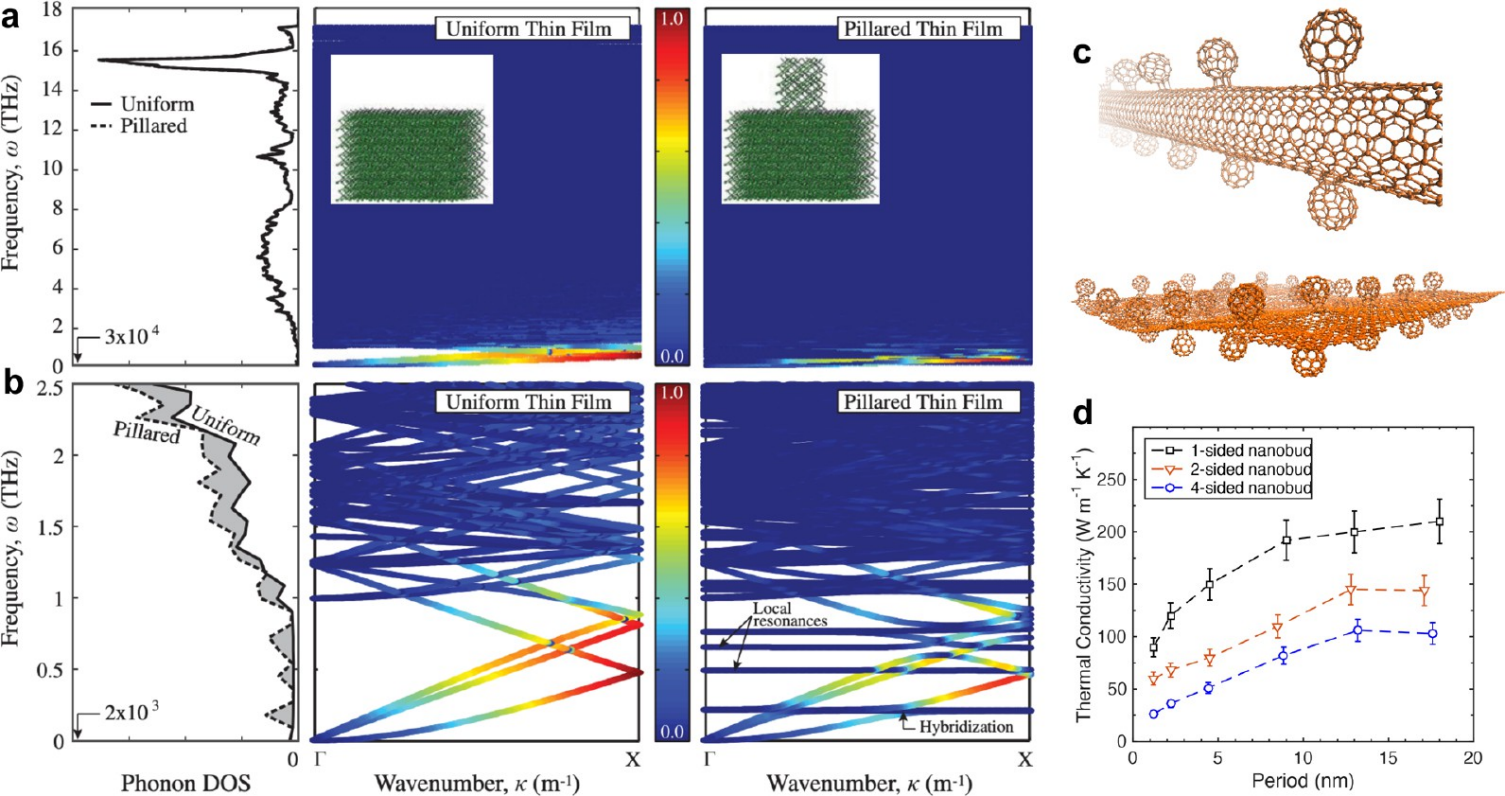
(a) Comparison of phonon density of states and phonon dispersion for (a) a silicon thin film and (b) a film with nanoresonators on the surface. Hybridized phonon states with dramatically reduced group velocities (and flat bands) appear as a consequence of resonance introduced by the vibrations of the pillars (taken from ref (104)). Adapted with permission from ref (104). Copyright 2014 American Physical Society. (c) Phonon resonance has also been shown for organic materials such as carbon nanotubes with fullerene functionalization (nanobuds) and graphene decorated with buckyballs.^105,106^ Adapted with permission from ref (105). Copyright 2018 American Physical Society. Adapted with permission from ref (106). Copyright 2019 American Institute of Physics. (d) Thermal conductivity can be engineered across a wide range by strategic placement of the fullerene resonators.^105^ Adapted with permission from ref (105). Copyright 2018 American Physical Society.

Another route to engineer heat conduction in solids through manipulating the wave-nature of phonons is via the inclusion of resonators on the surface of nanomembranes, thin films, and nanowires.^107^ This introduces hybridized modes in nanophononic metamaterials through phonon resonances in which a thermal conductivity reduction is realized via scattering of low-frequency (long mean free path) phonons rather than targeting nanoscale Bragg scattering as has been the usual strategy to manipulate heat conduction in nanostructured materials.^108^ Stated differently, the hybridization between resonant phonons and propagating modes of the underlying solid reduces the group velocities and phonon mean free paths of the low-frequency phonons that are otherwise hard to scatter with some of the often utilized nanostructuring techniques such as defect engineering, alloying, or inclusion of quantum nanodots, and as such, it is highly efficient in blocking phonon transport.^109^ This technique has been thoroughly demonstrated for resonators that are strategically placed on the surface of silicon thin films or nanowires,^109−112^ which introduces standing waves that hybridize with the underlying propagating modes giving rise to localized modes and avoided level crossings with greatly reduced group velocities (as evident from the flat bands in the phonon dispersion shown in Figure 4a and 4b for Si membranes taken from ref (104)). A similar coherent resonance effect has also been demonstrated in core–shell nanowires using MD simulations, where controlling the relative cross-section of the core and the shell regions can lead to the tunability in their thermal conductivities.^113^

While nanostructured inclusions in the form of quantum dots have been used to scatter phonons in solids, the uniqueness of the nanoresonators arises from the fact that they can manipulate waves with characteristic wavelengths that are several times larger than the characteristic dimension of the pillars (or the resonators). For the flat bands represented in the phonon dispersion, the mismatch in the vibrational amplitudes between the resonators and the underlying material causes only the atoms in the resonator to vibrate at that particular frequency. Such a concept has also been applied to organic materials based on carbon nanotubes and graphene sheets functionalized with strategic placement of fullerene molecules (Figure 4c) on the surface where localized resonances and modal hybridizations have been shown to remarkably lower their thermal conductivities by orders of magnitude (Figure 4d).^105,106^ Wang et al.^114^ have experimentally shown similar effects on the thermal transport of suspended trilayer graphene with gold nanoparticles deposited on the surface. They have shown that, with increasing surface coverage, the thermal conductivity decreases, which they attributed to the increased suppression of flexural acoustic phonon modes.^114^

The standing vibrations that couple across the boundary between the nanoscale resonators and the underlying structure can therefore result in (i) group velocity reductions, (ii) mode localizations, and (iii) reduction in lifetimes of vibrations across the entire broadband spectrum. This could prove to be very useful for engineering extreme thermal conductivity reductions in materials that are well suited for applications in thermoelectrics, which require uncompromised electronic and mechanical properties. In this regard, the general route to lowering the thermal conductivity of nanostructures through interfaces, grain boundaries, nanoinclusions, and pores can result in severely reduced electronic transport or mechanical integrity. Therefore, the main advantage of introducing nanoresonators on the surface to lower the thermal conductivity gives the opportunity to simultaneously adjust the electronic and thermal properties, all the while not influencing their mechanical integrity. For example, DFT calculations show that the electronic properties of nanobuds are highly tunable by changing the surface coverage of fullerenes on the sidewall of carbon nanotubes, while another work based on MD simulations shows that the surface coverage can tune the thermal conductivity through hybridization of vibrational states between the fullerenes and the underlying carbon nanotube (Figure 4d).^105,115^

Taken together, hybridized vibrational modes that couple across material interfaces provide a platform to control the thermal transport properties of nanocomposites and heterostructures without the need to intrusively modify their intrinsic microstructural architecture. These hybrid interfacial modes can also lead to emergent optical, electrical, magnetic, and thermal properties in superlattices, which can drive the pursuit of designer materials with unrivaled physical properties. However, these hybrid interfacial modes have only been studied in a handful of material systems, as mentioned in the above discussions. Therefore, although the aforementioned works have laid the foundation for such designer materials, there is still room for improving our understanding of hybrid vibrational modes in different types of nanocomposites to ultimately harness the potential of designer solids with extreme and tailored physical properties.

## III Hot Electron–Vibrational Coupled Energy Transport across Interfaces: Experiments and *Ab Initio* Simulations

The electron–phonon heat transfer mechanisms at both metal/metal and metal/non-metal interfaces have been historically robust topics of both theoretical and computational studies over the past several decades. Relatively few experimental works have studied these topics, presumably due to the rather well coordinated material systems and experimental parameters needed to properly examine these processes. Several recent reviews have focused on this electron–phonon energy exchange at interfaces under “near-equilibrium” conditions, which we define here as when the electrons and phonons in a metal can be described at the same temperature. For the most part, we focus this section on the current state of understanding of electron–phonon energy exchange across interfaces when the temperatures of these two subsystems are different. Often, this is denoted as “electron–phonon non-equilibrium”, which refers to the condition when the electrons are in local equilibrium (and can be well described by a Fermi–Dirac distribution and electron temperature) and the vibrations or phonons are in local equilibrium (and can be well described by a Bose–Einstein distribution and vibrational temperature), but these two temperatures are not equal. For the purpose of this Review, we avoid this terminology of “electron–phonon non-equilibrium” since this can often be muddled to also infer states of non-equilibrium in the electron subsystem (i.e., strongly non-Fermi), a topic that has been studied in depth, especially in the context of short pulsed laser heating.

Thus, we focus our discussion in this section on electron–phonon or electron–vibrational coupled energy transport processes across interfaces when the electron and phonon/vibrational subsystems are both well described by only slight perturbations from their respective equilibrium distributions, but each of their equilibrium distributions are defined by statistically and significantly different temperatures. We refer to this state of a material as electron–phonon (EP) or electron–vibrational (EV) coupled local equilibria (CLE). Our discussion below begins by reviewing the current state of experimental measurements of EP heat transfer processes at metal/metal interfaces followed by metal/non-metal interfaces during conditions of CLE. We culminate the experimental section by reviewing a recent experimental observation of ballistic thermal injection (BTI) at metal/doped non-metal interfaces that relies on EP CLE between the metal electrons and electrons and phonons in the doped non-metal. This BTI process was used to create long-lived hot electrons in the non-metal to control plasmonic absorption, offering an approach to thermally modulate plasmon resonances and thus optical absorption in non-metals. Further, BTI can be used to transiently control the directionality of heat flow across metal/non-metal interfaces, unlocking potential avenues for ultrafast thermal diodes that rely on EP CLE.

True quantum mechanical insight into the energy exchange mechanisms associated with hot electrons in CLE with the phonons and vibrations at interfaces comes from *ab initio* quantum dynamics simulations.^116−118^ This approach provides a fundamental perspective on the evolution of hot carriers coupled to vibrational motions by creating a time-domain atomistic description, most closely mimicking the time-resolved experiments. We conclude this section with a review of recent advances in *ab initio* quantum dynamics simulations that have focused on EP and EV CLE at metal/metal and metal/non-metal interfaces, including the role of plasmon-like states on interfacial energy exchange.

### III.a Hot Electron–Phonon Heat Transfer Processes at Metal/Metal Interfaces

Under near-equilibrium conditions, when the electrons and phonons in a metal can be described by the same temperature, the thermal boundary conductance at metal/metal interfaces can be well captured by the ratio of the electronic density of states and Fermi velocities in the materials on either side of the interface.^119,120^ This theory is rooted in the diffuse mismatch model for the electrons that has been confirmed experimentally and follows the Wiedemann–Franz law at interfaces.^121,122^ However, we note that this has not been studied in metals with relatively high phonon contributions to heat conduction, such as for tungsten.^123^

Under conditions of EP CLE, Hopkins et al.^124^ predicted that, in the absence of electron–phonon scattering, the electron–electron thermal boundary conductance is also driven by the differences in the electronic densities of states of each material adjacent to the interface, similar to the near-equilibrium scenario discussed above. Under conditions of strong EP CLE and high electron temperatures, the electron–electron thermal boundary conductance was predicted to deviate from the free electron predictions based on changes in the electron density of states within a few *k*_B_*T*_e_ of the Fermi level. At these extremely elevated electron temperatures, electron–electron thermal boundary conductances were predicted that far exceed 10 GW m^–2^ K^–1^,^124^ values that are orders of magnitude higher than typical phonon–phonon dominated thermal boundary conductances. However, these model predictions of electron–electron thermal boundary conductance at metal/metal interfaces during strong EP CLE have never been experimentally validated. To do so would involve the use of a short-pulsed pump–probe experiment that could accurately determine the metal/metal thermal boundary conductance at electron temperatures of thousands of Kelvin before any electron–phonon scattering occurs, so that the phononic subsystems of each metal at the interface remain cold. This extremely high electron–electron thermal boundary conductance at metal/metal interfaces during electron–phonon non-equilibrium could have a tremendous impact on heat removal during metal 3D printing and short-pulsed manufacturing.

At time scales during and after EP CLE at metal/metal interfaces, Qiu and Tien predicted that the interfaces can lead to subsurface and delocalized heat sinking depending on the thickness of the top metal and strength of electron–phonon coupling.^125^ Specifically, they theoretically examined the electron and phonon heat transfer processes in laser irradiated Au, Au/Cr, and Au/Cr/Au films. The weak electron–phonon coupling in Au results in longer lived hot electrons in the excited Au that traverse across the Au/Cr interface and couple their energy to the Cr phonons before the Au phonons heat up. This results in subsurface heating, where, for a few picoseconds, the Au lattice remains cold, while the Cr lattice under the Au surface is hot. This indirect heating was described in an analogy by the late Professor Chang-Lin Tien as being akin to “staring at the sun and getting sunburned on the back of your head”^126^ and has been since observed in transient temperature rises monitored during ultrafast laser heating of metallic bilayers and metal thin films with adhesion layers on insulating substrates.^127,128^ The ultrafast interfacial heat transfer processes and subsurface heating of laser excited metallic bilayers under conditions of EP CLE were later experimentally studied in a series of works that used this geometry to accurately deconvolve ballistic heat transfer in self-assembled monolayers,^129,130^ thermally induced spin current and torque,^131,132^ robust measurements of the intrinsic electron–phonon coupling factor in Au and Cu,^133^ subsurface melting in deeply undercooled silver,^134,135^ and measurements of the ballistic electron–phonon mean free path in metals^136^ and to assess the role of oxygen defects and adhesion layer stoichiometry on the electron–electron thermal boundary conductance across Au/TiO_*x*_ interfaces.^136^

### III.b Hot Electron–Phonon Heat Transfer Processes across Metal/Non-Metal Interfaces

**Figure 5:**
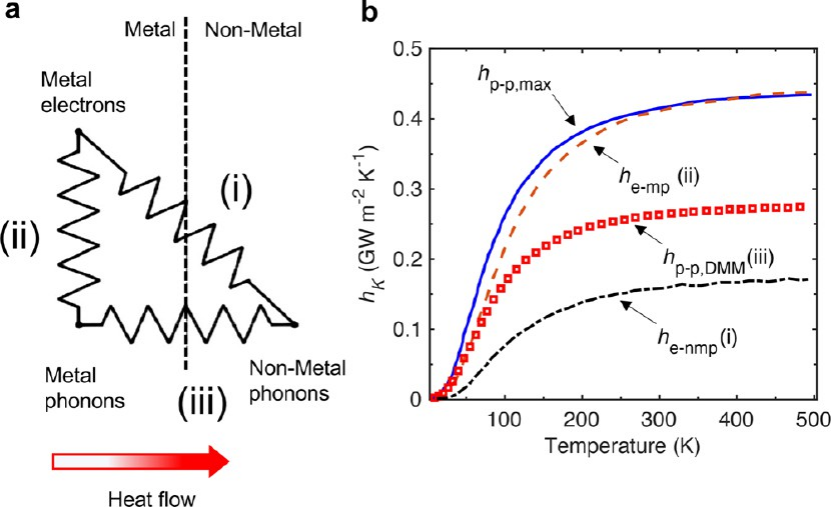
(a) Schematic representation of various pathways for metal/non-metal interfacial heat conduction. The electronic subsystem coupling with the lattice in the bulk of the metal presents a resistance that is in series with the purely phonon-driven coupling across the metal/non-metal interface. However, the alternate channel where the electrons can directly transfer their energy across the interface to the non-metal lattice could also affect interfacial heat conduction.^50^ Adapted with permission from ref (50). Copyright 2004 American Institute of Physics. (b) Interfacial conductance across the TiSi_2_/Si interface influenced by the various channels of heat flow. Adapted with permission from ref (137). Copyright 2015 American Institute of Physics. The thermal boundary conductance from channel (i) where the electrons directly couple to the phonons in the non-metal is on the same order of magnitude as that for the purely phonon-driven conductance as described by channel (iii).

At metal/non-metal interfaces, the processes that contribute to thermal boundary conductance during EP CLE are more obfuscated compared to the metal/metal interface case. Relative to metal/metal interfaces, the role of electron–phonon coupling on the thermal transport across metal/non-metal interfaces is less clear due to the lack of direct experimental evidence proving or disproving the influence of this coupling mechanism, regardless of the degree of non-equilibrium. The pathway of electron–phonon coupling at metal/non-metal interfaces under near-equilibrium transport conditions (i.e., when the electrons and phonons in the metal can be described by a similar temperature) has been theorized to influence thermal boundary conductance via two pathways, depicted in Figure 5a. Unlike at metal/metal interfaces, at metal/non-metal interfaces, the negligible number density of free electrons available in the non-metal leads to the electron–electron thermal boundary conductance becoming a nonexistent heat transfer pathway, consistent with the diffuse mismatch model for electrons described above. In the case that the non-metal is doped to increase the free electron number density, this electron–electron thermal pathway can begin to contribute to thermal conductance,^138^ which we describe in more detail later in this section. Given this, the three assumed mechanisms for electron–phonon coupling heat transfer at metal/non-metal interfaces are (i) electrons in the metal coupling to phonons in the non-metal across the metal/non-metal interface, (ii) electrons in the metal coupling to phonons in the metal on the metal side of the metal/non-metal interface, and (iii) subsequent phonon–phonon conductance across the interface. We focus our discussion here on mechanism (i), since this represents heat transfer across an interface. As originally derived by Majumdar and Reddy,^50^ mechanism (ii) involves electron–phonon coupling in the metal and heat transfer across the metal/non-metal interface is still driven by phonon–phonon coupling (iii); thus, we do not focus our discussion on these mechanisms here. However, recent reviews have discussed this process in detail,^42,62^ and we refer the readers to these extensive bodies of work for an in-depth review of this process. We therefore focus our discussion on the current state of understanding of the mechanisms of metal electron to non-metal phonon coupling across a metal/non-metal interface, specifically during CLE.

We begin this discussion with the current state of understanding of the metal electron to non-metal phonon heat transfer processes during near-equilibrium conditions. This process was quantum mechanically theorized by Sergeev^51,52^ to be driven by electronic scattering processes similar to electron-defect scattering. Additional analytical theories by Huberman and Overhauser^139^ and Mahan^140^ suggest that this mechanism is driven by metal electrons interacting with different types of interfacial states at the metal/non-metal interfaces. Lu et al.^141^ have argued that surface state electrons from the metal side can couple with the phonons of the semiconductor or the insulator on the other side, which should be considered in parallel with the phonon–phonon heat conduction channel across the metal/non-metal interfaces. More recently, Sadasivam et al.^137^ used an *ab initio* approach to determine that the electron–phonon coupling at the interface between a metal silicide and silicon can lead to electron–phonon thermal boundary conductances that can rival that of phonon–phonon thermal boundary conductances (Figure 5b). Furthermore, through the non-equilibrium Green function method, Zhang et al.^142^ have shown that thermal rectification can be achieved in metal/non-metal interfaces (for their simplified one-dimensional model) due to the vastly different energy carrier populations on either side (namely, electrons in the metal side and phonons in the non-metal side) of the interface. This topic of thermal rectification was raised as early as 1969 by Pollack^143^ in his seminal review, and as shown by Li et al.,^144^ asymmetric thermal boundary conductance can be achieved when highly dissimilar materials come in contact (where phonons from one side can couple with drastically different heat carrier populations). However, we will not review this topic here but refer the interested readers to refs (145) and (146) for a detailed discussion regarding thermal diodes and thermal rectification.

Under near-equilibrium conditions between electrons and phonons, various experimental works have ruled out that this mechanism will play a role in thermal conductance based on empirical evidence of measured thermal boundary conductances across various metal/non-metal interfaces composed of different metals with widely varying electronic densities of states at the Fermi level or different electron–phonon coupling strengths,^38,128,147^ or by comparing measured thermal boundary conductances across high-quality interfaces to rigorous phonon transport calculations.^148^ An additional work by Ye et al.^149^ suggested that this metal electron to non-metal phonon interfacial interaction may be playing a role at metal silicide/silicon interfaces but could not conclusively rule out other possibilities. However, this work did conclusively rule out that electron–electron interactions were playing a role even for carrier concentrations in the silicon of up to 10^19^ cm^–3^.^149^

This segues to the question: do these electron–electron and electron–phonon interfacial heat transport mechanisms at metal/non-metal interfaces influence thermal boundary conductance during conditions of CLE when the electrons are hotter than the phonons? Giri et al.^150^ developed a coupled thermodynamic and quantum mechanical deviation of electron–phonon scattering at free electron metal/non-metal substrate interfaces using Fermi’s golden rule coupled with diffuse mismatch theory to predict the thermal boundary conductance between metal electrons and non-metal phonons when the hot metal electrons can be at elevated temperatures as compared to the non-metal phonons. Under near-equilibrium conditions this theory agreed well with predictions using Sergeev’s model.^51,52^

Even compared with the relatively sparse collection of studies that have focused on the near-equilibrium situation, the CLE body of work has been even less studied, offering the potential for robust increases in our understanding of this process moving forward with formed experimental studies. These model predictions of thermal boundary conductance at metal/non-metal interfaces during strong EP CLE would involve the use of a short-pulsed pump–probe experiment that could accurately determine the metal/non-metal thermal boundary conductance at electron temperatures of thousands of Kelvin before any electron–phonon scattering occurs so that the phononic systems of each metal at the interface remain cold. Several groups have noted that hot electron scattering at a metal/non-metal interface can lead to enhancements in the overall electron–phonon equilibration of a metal,^151−153^ although the atomistic mechanism in which this equilibration is enhanced was not studied and thus the question remains if this metal electron to non-metal phonon heat transfer pathway was driving these observations. Giri et al.^128^ and Olson et al.^136^ observed enhancements in hot electron equilibration in Au films with Ti adhesion layers that varied based on the substrate or Ti oxygen stoichiometry, respectively, and subsequent *ab initio*-based simulations revealed that this mechanism was most likely driven by enhanced electron–phonon coupling in the Ti and additional electron-defect scattering in the Ti layer. The key to understanding this mechanism was the development of *ab initio* quantum dynamics simulations, which provides a fundamental perspective on the evolution of hot carriers coupled to vibrational motions and can be applied to atomic heterojunctions. We review this recently developed approach by the Prezhdo group, which combines real-time time-dependent DFT for the evolution of the electrons with non-adiabatic molecular dynamics for the evolution of ionic cores and electron–vibrational interactions in Section III.d. With this, the metal hot electron to non-metal phonon direct interaction and resulting thermal boundary conductance has still yet to be directly experimentally observed.

### III.c Ballistic Thermal Injection (BTI)

**Figure 6:**
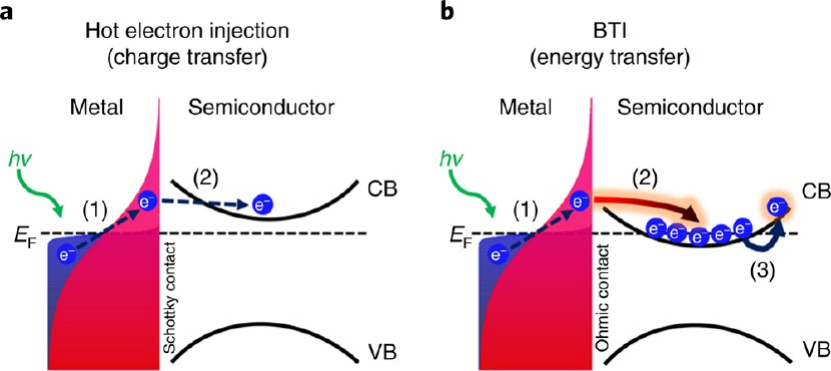
(a) Hot electron injection: the process typically assumed to occur at metal/semiconductor interfaces after photoexcitation of the metallic contact. In this case, hot electrons are first generated in Au (1). At sufficiently high electron temperatures, the electrons traverse the interface and add charge to the conduction band (CB) of the semiconductor (2). (b) Ballistic thermal injection (BTI): our proposed process for energy transfer across metal/semiconductor interfaces after an ultrafast excitation of the metal contact. This mechanism relies on hot-electron generation in the metal (1); prior to the electron–phonon coupling (less than a couple of picoseconds), energy propagates ballistically toward the metal/semiconductor interface. The electron energy front reaches the interface, whereby the electrons transfer their energy (2), rather than charge, to the pre-existing free electrons in the semiconductor’s conduction band. The pre-existing semiconductor’s electrons are now at an elevated temperature. Adapted with permission from ref (138). Copyright 2021 Springer Nature.

A CLE process between hot electrons in a metal and electrons and phonons in a doped non-metal was recently observed by Tomko et al.^138^ at Au/doped CdO interfaces, a process deemed “ballistic thermal injection” (BTI). Ballistic thermal injection (BTI) is a recently discovered energy transduction mechanism that arises from the CLE dynamics at metal/non-metal interfaces. In short, BTI is an interfacial energy injection mechanism (without concomitant charge injection) that was observed at an interface between ultrafast laser-excited Au- and Y-doped CdO. When electrons in the Au are excited from the sub-picosecond laser pulse, they travel ballistically toward the metal/non-metal interface; upon reaching the interface, these hot electrons in the metal scatter with electrons in the non-metal, the heat transfer of which is facilitated by the high electron-mediated thermal boundary conductances discussed in Section III.b, resulting in enhanced transmission of energy into the non-metal. The key is that this energy flow is facilitated by the ballistic impingement of electrons at the interface and not diffusive phonon energy transport. This facilitates energy movement across the interface with an efficiency orders of magnitude higher than in typical diffusive transport regimes. Since the energy from the hot electrons in the Au is transferred to the free electrons in the doped CdO, the electrons in the CdO are now at a different temperature than the phonons in the CdO, and this CLE exists in the CdO that was initiated from BTI originating from the hot electrons in the Au. This process is fundamentally different than hot electron injection, and a comparison of these two processes is shown in Figure 6.

In principle, this BTI process can occur at any metal/doped non-metal interface with high enough carrier concentrations. For example, the previously studied metal/metal-silicide interface by Ye et al.^149^ could be a candidate to observe BTI given hot metal silicide electrons in CLE with the phonons. However, there could also be a material constraint on this BTI process being a significant contributor to thermal boundary conductance. CdO and doped metal oxides in general offer a distinct advantage for enhancing BTI due to their ability to maintain high electron mobilities at carrier concentrations as high as 10^19^–10^20^ cm^–3^.^154,155^ However, this process has only begun to be studied, and thus, additional investigations into different material interfaces and the role of CLE on enhancing this BTI process are warranted and would result in hot-electron-based control over various material functionalities. For example, in the work from Tomko et al.,^138^ they used this BTI process to control the infrared plasmonic response of CdO via ultrafast thermal modulation of CdO’s epsilon near zero mode.

Additionally, one can envision the ability to create delocalized thermalization and transient thermal diodes by embracing BTI at metal/semiconductor interfaces. In this process, the non-metal electrons, after gaining energy from the high energy density electrons from the metal, then conduct heat away from the interface based on the electron mobility in the non-metal. The electrons in the non-metal then thermalize with the phonons in the non-metal, creating the hot-spot from this BTI process that is spatially removed from the metal interface, akin to the metal/metal discussion in Section III.a. While some thermal energy leaks back into the metal, this process is relatively slow and inefficient relative to BTI, since this process of energy being “re-deposited” into the metal after thermalization is diffusive and thus relies on the relatively low phonon–phonon thermal boundary conductance at the metal/non-metal interface. In this sense, harnessing BTI allows for thermal energy to be more effectively dissipated via ballistic mechanisms in one direction than via diffusive mechanisms in the reverse direction, thus enhancing cooling of hot spots via a thermal diode effect across interfaces.

### III.d *Ab Initio* Electron–Vibrational Dynamics at Metal/Semiconductor Interfaces

As overviewed in Section III.b, several theoretical approaches have been applied to study the coupled heat transfer mechanisms at the interface between electrons in metals and electrons and phonons in semiconductors. The key in advancing our understanding of these processes and interpreting experimental measurements lies in accurately determining the electronic and phononic bandstructure of the metal, semiconductor, and its interface, as astutely pointed out by Sadasivan et al.^137,156^ Subsequently, using the proper framework, various excited energy and/or higher temperature states in the electronic systems can be modeled to capture the CLE between the electrons and vibrational states and resulting energy exchange and thermal boundary conductance across the metal/non-metal interfaces. Sadasivan et al.^137,156^ suggested this could be accomplished with the non-equilibrium Green function (NEGF) formalism, a well-developed approach to model transport in electron and phonon systems with atomistic control and input.^157,158^ Here, we review a recently developed approach, rooted in *ab initio* quantum dynamics simulations, that captures the electron–vibrational CLE dynamics at metal/semiconductor interfaces by combining non-adiabatic molecular dynamics and time-dependent DFT. This robust technique not only allows for the degree of CLE between electronic and vibrational states to be controlled but also can be extended to capture plasmon dynamics and resulting energy exchange at metal/non-metal interfaces, offering insight into the energy exchange processes during CLE.

#### III.d.1 Non-Adiabatic Molecular Dynamics and Time-Dependent Density Functional Theory

*Ab initio* quantum dynamics simulations^116−118^ provide a foundational perspective on the evolution of hot carriers coupled to vibrational motions, by creating a time-domain atomistic description, most closely mimicking the time-resolved experiments. The state-of-the-art methodology, developed by the Prezhdo group,^159,160^ combines real-time time-dependent DFT for the evolution of the electrons with non-adiabatic molecular dynamics for the evolution of ionic cores and electron–vibrational interactions. The electrons are treated quantum mechanically by solving the time-dependent Schrodinger equation, which depends parametrically on the classical vibrational coordinates. The vibrational motions are described classically by molecular dynamics with semiclassical corrections.^161^ The electron–vibrational coupling matrix elements are computed on-the-fly along the trajectories. Charge–charge scattering is described by screened Coulomb interactions.^118,162^ Quantum transitions between electronic states are modeled by surface hopping,^116,117,159^ which can be viewed as a master equation with non-perturbative configuration- and time-dependent transition rates.^116,163,164^

#### III.d.2 Excitations in Metallic Structures and Electron–Vibrational Interactions

**Figure 7:**
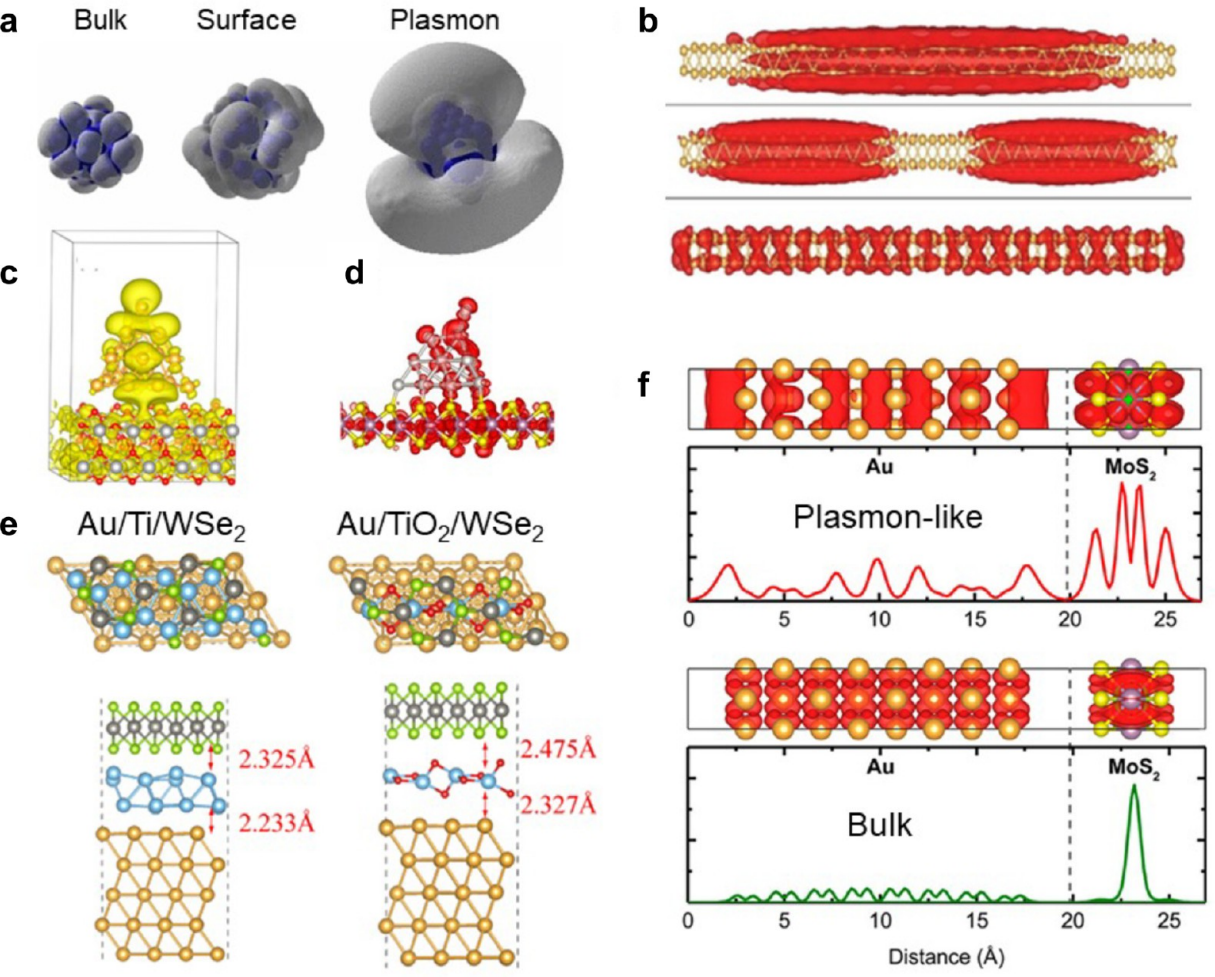
Examples of metallic systems studied by *ab initio* quantum dynamics simulation. (a) Bulk, surface, and plasmon states in a Ag nanoparticle. The plasmon states extend significantly outside the atomic structure and couple to vibrations much more weakly than the bulk and surface states. Adapted with permission from ref (166). Copyright 2010 American Physical Society. (b) Plasmon-like (top and middle) and regular (bottom) excitations in a Au nanorod.^183^ Adapted with permission from ref (183). Copyright 2018 Elsevier. (c) Plasmon resonance in a Au pyramid on a TiO_2_ surface. The charge density (yellow) is largest on the pyramid apex; however, charge density is significantly delocalized onto the TiO_2_ substrate, leading to a charge-transfer plasmonic excitation. Adapted with permission from ref (173). Copyright 2014 American Chemical Society. (d) Pt pyramid on a MoS_2_ monolayer. The top atom of the pyramid fluctuates away from the equilibrium location on a 100 ps time scale, giving rise to a longer lived resonance, whose density is shown in red. Adapted with permission from ref (174). Copyright 2020 American Chemical Society. (e) WSe_2_/Au interface with Ti (left) and TiO_2_ (right) adhesion layers. Ti provides strong adhesion, accelerating energy transfer across the interface. TiO_2_ gives weaker adhesion and can cause defects, such as a switch of the O and Se atoms. (f) Plasmon-like states (top) extend far into the space between Au and MoS_2_, giving rise to a slow decay of the charge transfer rate with the Au–MoS_2_ distance. The charge transfer rate decays much faster at lower energies, at which only bulk excitations are possible (bottom). Adapted with permission from ref (186). Copyright 2021 American Chemical Society.

Metals support several types of electronic excitations, including single particle, image-potential, and collective states.^165^ Parts a and b of Figure 7 provide examples of bulk, surface, and plasmon-like states in metallic nanoparticles. Bulk states are localized within a metal, surface resonances are supported by unsaturated chemical bonds, and plasmonic excitation density extends far outside the material. Electron–vibrational interactions are significantly weaker for electronic states localized away from the atoms,^166^ since the coupling matrix element depends on the sensitivity of the electronic states to atomic displacements.

Electron–vibrational interactions produce elastic and inelastic scattering. Inelastic scattering involves the exchange of electronic and vibrational energy, while elastic scattering induces the loss of phase information and coherence. The decoherence time can be quantified as the pure-dephasing time,^167^ whose inverse gives the homogeneous optical line width. Classic theories of surface plasmons consider plasmon–surface scattering to be the fastest contribution to plasmon dephasing.^168^ Since plasmonic excitation is localized away from atoms, the electron–phonon coupling is much weaker for plasmons than bulk and surface states, as demonstrated for Ag clusters using *ab initio* molecular dynamics.^166^ Phonon-induced pure-dephasing of plasmons took 30–40 fs and showed modest size dependence, with electron–phonon coupling being stronger in smaller clusters. Both plasmon and non-plasmon states coupled mostly to low-frequency acoustic phonons that modulated the size and shape of the nanoparticles and were sensitive to temperature variations, because the frequencies of acoustic phonons in metallic clusters are comparable to the thermal energy, *k*_B_*T*. The inelastic scattering process in a Ag particle took picoseconds,^169^ 2 orders of magnitude longer than the elastic scattering. Higher energy plasmon excitations were delocalized farther away from the cores and, hence, exhibited weaker coupling to phonons and decayed more slowly than lower energy plasmons. The picosecond lifetimes of hot electrons generated by plasmon excitations can be sufficient to allow charge extraction or elementary photochemical events associated with bond breaking, formation, or rearrangement.

The elastic and inelastic electron–phonon scattering analyses were performed on Au films,^170^ rationalizing the time-resolved thermo-reflectance measurement.^128^ Increasing temperature accelerated both processes and allowed a broader range of vibrations to couple to the electronic excitations due to increased anharmonicity. The inelastic scattering was strongest between states with small energy differences and the elastic scattering was fastest between pairs of states that were distant in energy, because it is determined by the magnitude of the phonon-induced energy gap fluctuation, which generally increases for larger gaps.^167^

#### III.d.3 Special, Longer-Lived Electronic States

Metals exhibit a variety of structural motifs on the surface, and certain structures can give rise to states that partly decouple from the continuum of bulk states and trap charges for relatively long times. This is particularly important for catalysis, which often occurs on defects, single metal atoms, or metal clusters. Hot electron relaxation in the Au_55_ cluster showed a long-lived intermediate state 0.8 eV above the Fermi level.^171^ The long lifetime of the intermediate state facilitated the charge transfer to the TiO_2_ substrate. Interestingly, the Au_55_/TiO_2_ and Ru_10_/TiO_2_ systems showed picosecond electron–vibrational relaxation,^171,172^ while the Au_20_/TiO_2_ exhibited sub-picosecond relaxation.^173^ The difference could be linked to the cluster shape; both Ru_10_ and Au_55_ were spherical, while the Au_20_ particle was a pyramid.

A machine learning force field was developed to study excited state quantum dynamics in the pyramidal Pt_20_ cluster adsorbed on the MoS_2_ substrate.^174^ While the perfect Pt_20_ pyramid gave rapid electron–phonon relaxation, the top atom of the pyramid was oscillating between two configurations on a hundred picosecond time scale, producing another structure (Figure 7d). Partially detached, the top atom supported a localized electron trap with reduced coupling to the rest of the system. The lifetime of the special state was 3 times longer compared to that of the perfect pyramid, creating favorable photocatalytic conditions. Whether or not the trap state was populated depended on the initial energy of the electron as it entered the Pt_20_ particle. Higher energy initial states had access to additional relaxation pathways, bypassing the trap state. The structural distortion of Pt_20_, supporting a longer-lived electron trap, provides a scenario that may play a key role in metal cluster catalysis driven by hot electrons.

#### III.d.4 Substrates Can Have a Strong Influence on Charge Carrier Dynamics in Metals

Substrates and adhesion layers are commonly used to assemble complex nanostructures to achieve desirable optical and electronic functionalities. The electronic response of metal/substrate composites can differ from that of isolated structures. Interaction between a metal and a conducting substrate may give rise to composite plasmon–plasmon resonances.^175^ Inclusion of adhesion layers,^176^ surface adsorbates,^47^ and molecules^177^ between metal films and semiconducting or dielectric substrates modulates charge and energy flow across the interface. Thin Ti and TiO_*x*_ films have been used as adhesion layers between Au films and dielectric substrates to improve thermal transport (Figure 7e). Experiments showed that inclusion of a Ti layer enhanced the interfacial interaction and accelerated the energy flow,^128^ and *ab initio* quantum dynamics simulation^178,179^ identified the mechanism. First, the Ti layer greatly enhanced the density of states in the relevant energy window. Second, Ti atoms were much lighter than Au atoms, creating strong electron–phonon coupling. Further calculations^180^ demonstrated that the conclusion was robust to partial alloying between the Ti adhesion layer and the Au films.

TiO_*x*_ layers exhibit various stoichiometries, with *x* ranging from 0 to over 2. The quantum dynamics calculations^181^ demonstrated the TiO_*x*_ chemical composition strongly influenced the electron–vibrational relaxation. The effect was related to the energy alignment between Au and TiO_*x*_ and to the strength of interfacial bonding. Oxygen-rich and oxygen-poor TiO_*x*_ could be used to control the hole and electron relaxation, respectively. The theoretical prediction was verified experimentally^136^ and applies generally to other systems.

#### III.d.5 Charge Transfer at Metal/Semiconductor Interfaces

Plasmons exhibit very strong optical activity, and charges photogenerated by a plasmonic excitation can be extracted by semiconductors to create photovoltaic and photocatalytic devices. The conventional model involves the indirect plasmon-induced hot-electron transfer (PHET) mechanism by which hot carriers are initially generated in the metal by plasmon decay and then transfer to an acceptor. However, PHET is rather inefficient, since hot electrons in a metal have short lifetimes. An alternative proposal was put forth theoretically^173^ based on the quantum dynamics simulation. The experimental demonstration came a year later.^182^ The direct plasmon-induced charge transfer (PICT) mechanism relies on a strong metal–semiconductor interaction due to which the charge density of the plasmonic excitation attains a strong tail extending into the acceptor (Figure 7c). Upon plasmonic excitation, an electron appears inside the semiconductor instantaneously with a significant probability, around 25% in the considered cases.^173,182^ PICT bypasses fast electron–vibrational relaxation inside the metal and, therefore, increases the charge transfer efficiency.

The initial theoretical^173^ and experimental^182^ work demonstrating PICT focused on interfaces of metallic particles with 3D semiconductors, the surfaces of which contain unsaturated chemical bonds that create strong interfacial coupling. Interaction of metals with 2D materials is weaker because 2D materials contain no dangling chemical bonds. Therefore, the traditional PHET mechanism plays a more important role with 2D substrates.^183^ The two mechanisms, PHET and PICT, can coexist even in weakly bound hybrids.^184^ The combination of the two mechanisms leads to fast charge transfer, overcoming hot-carrier cooling. Spatial polarization of the excited state in the metal influences its coupling to the semiconductor and alters the contributions of each mechanism. The probabilities of PHET and PICT are also sensitive to external stimuli, such as strain.^185^

#### III.d.6 Energy Transfer at Metal/Semiconductor Interfaces by Electron–Electron Scattering

Hot electrons in a metal can transfer energy across the metal/semiconductor interface without transferring charge. Electron–vibrational relaxation can heat up phonons in a metal, and then, metal phonons can scatter with phonons in a semiconductor. Alternatively, hot electrons in a metal can scatter with charges in the semiconductor that is intrinsically or extrinsically doped. A sufficiently high concentration of charge carriers in the semiconductor is required to transfer significant amounts of energy. However, the energy transfer via charge–charge scattering is much faster than the transfer via phonon–phonon scattering. The interaction occurs via Coulomb coupling,^118^ and the *ab initio* simulation requires consideration of ensembles of electrons.^164^ If the thickness of the metal layer is less than the length of electron scattering inside the metal, then the energy transfer across the metal/semiconductor interface becomes ballistic and extremely fast. Such a process has been observed and modeled at the Au/CdO interfaces (Figure 6).^138^ Typically, one expects a rapid decay of the charge transfer rate since the tunneling barrier becomes increasingly wider.

#### III.d.7 Plasmon-Like States Can Accept Charges from Semiconductors

Most of the examples discussed above focused on the quantum dynamics of hot electrons generated in a metal. The opposite process, in which hot electrons generated in a semiconductor transfer charge or energy into a metal, is also important from both fundamental and applied points of view. The joint experimental and theoretical study^186^ uncovered an unusually efficient charge transfer from MoS_2_ into a Au film over large spatial gaps (Figure 7e). Charge transfer events were measured with both spatial and temporal resolution, and charge transfer rates were studied as a function of the distance between MoS_2_ and Au. However, the measured charge transfer rate decayed unusually slowly with the MoS_2_/Au separation,^186^ as is typical of charge transfer facilitated by conducting molecular bridges. After a critical distance, the rate dropped fast, as expected for the tunneling barrier. Calculations rationalized this observation by the participation of plasmon-like acceptor states in the metal. These states extend many angstroms beyond the metal, bridging the semiconductor/metal gap (Figure 7f, top panel). The weak sensitivity of the charge transfer process on the donor/acceptor separation suggests that it should be robust to surface corrugation and defects, a significant benefit for optoelectronic devices.

## IV Photon–Phonon Energy Transduction

In this section, we summarize the recent research involving surface phonon polaritons (quasiparticles composed of strongly coupled photons and optical phonons) for thermal radiation and thermal conduction at surfaces and interfaces. For polar materials, such as SiC, SiO_2_, and hBN, the coherent vibrations of the lattice (optical phonons) result in a net dipole moment, leading to the strong absorption of IR light at the transverse optic (TO) phonon frequency. The corresponding longitudinal optic (LO) phonon is shifted to higher frequencies due to the breaking of the degeneracy of the optical phonons at the Γ point. This spectral splitting gives rise to the so-called Restrahlen band where the real part of the permittivity tensor becomes negative. This is the critical requirement for stimulating surface phonon polaritons (SPhPs) at an interface between the polar crystal and a dielectric (typically air). SPhPs result in highly confined optical fields and substantially reduced resonance line widths due to the long lifetime of the optical phonons (on the order of a few to tens of picoseconds) in comparison to their plasmon polariton counterparts.^187,188^ However, there is a large momentum mismatch between these SPhPs in the energy–momentum dispersion relationship with light; i.e., SPhPs cannot be stimulated directly from free-space photons. To overcome this mismatch, high-index prisms, gratings, nanostructures, and sub-wavelength scatterers such as the metallized atomic-force microscope tip employed in scattering-type scanning near-field optical microscopy (s-SNOM) can be employed to launch and probe SPhPs.^56^ While metals usually exhibit isotropic dielectric functions, low-symmetry polar crystals can possess strongly anisotropic permittivity values along three optical axes. The anisotropic dielectric functions of a polar material can even go to the extreme such that at least one of the principal components of the permittivity has the opposite sign with respect to the other principal components. This leads to only certain directions being allowed for SPhP propagation according to the hyperbolic isofrequency contours (IFCs),^189,190^ for example, out-of-plane hyperbolic phonon polariton propagation in hBN,^191^ in-plane hyperbolic phonon polariton propagation in α-MoO_3_^192^ and V_2_O_5_,^193^ ghost polariton propagation in calcite (CaCO_3_),^194^ and hyperbolic shear polaritons in beta-phase Ga_2_O_3_ (bGO).^195,196^ The above optical properties for polar crystals present opportunities to engineer the radiative heat transfer (both in the far-field^53,197−199^ and near-field^200−202^) and potentially conductive thermal transport via SPhP stimulation.^203,204^

**Figure 8:**
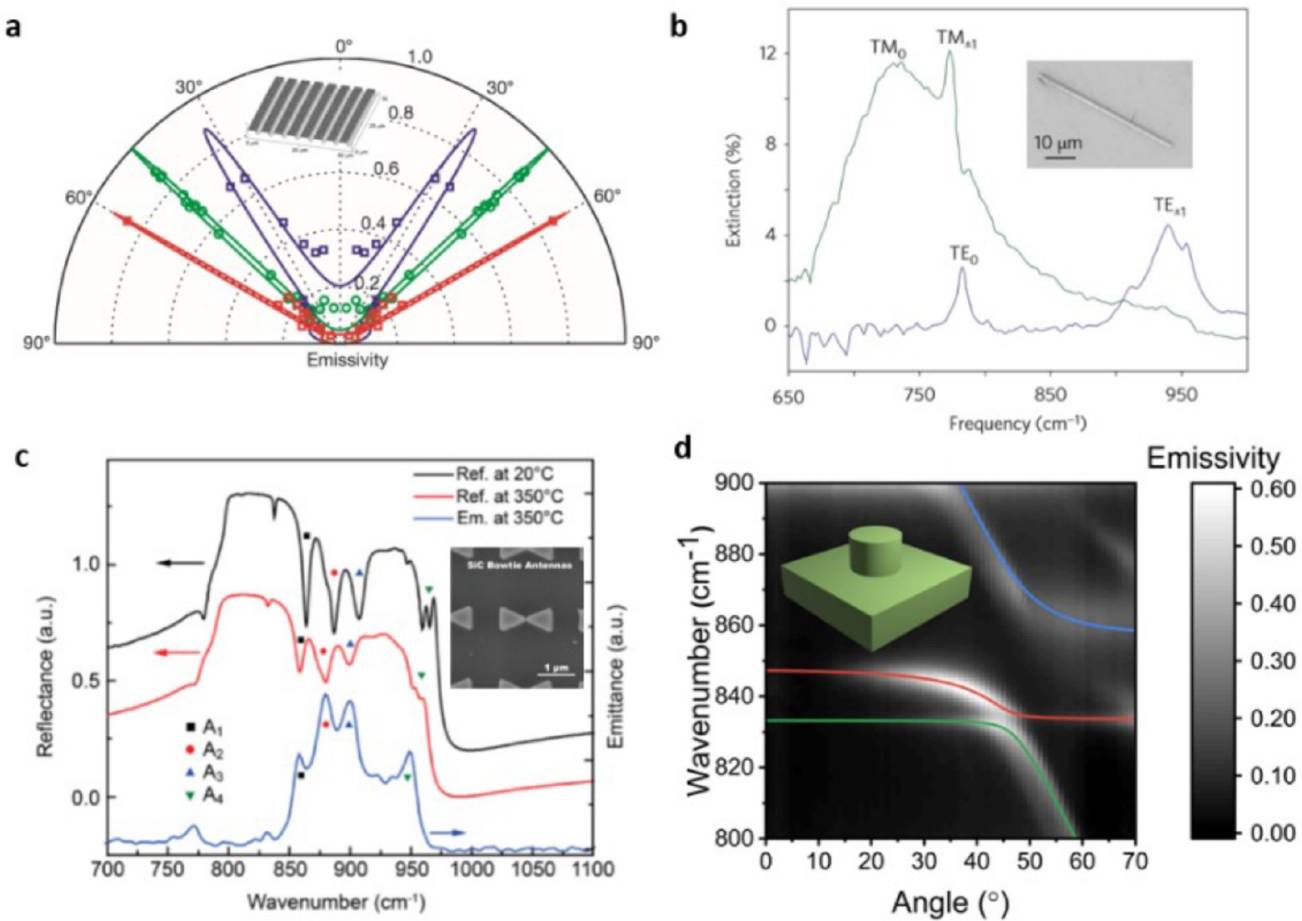
Far-field thermal emission engineering using surface phonon polaritons. (a) Directional thermal emission from a 1D SiC grating (an AFM image of the SiC grating is shown in the inset). Different colors represent different emission frequencies with the emission angles following the SPhP dispersion relationship. Adapted with permission from ref (197). Copyright 2002 Springer Nature. (b) Polarized thermal emission spectra from a single cylindrical SiC antenna (shown in the inset). Adapted with permission from ref (53). Copyright 2009 Springer Nature. (c) Narrowband thermal emission from periodic arrays of SiC bowtie nanoantennas (SEM image of the bowtie array shown in the inset). The blue line is the measured thermal emission spectrum at 350 °C, which is compared with the reflection spectrum at room temperature (black line) and at 350 °C (red line). Adapted with permission from ref (198). Copyright 2017 American Chemical Society. (d) Measured angular thermal emission plot from a strongly coupled thermal emitter made of SiC nanopillars (the unit cell is shown in the inset). Through the introduction of strong coupling between the propagating (blue line) and localized SPhPs modes (red line) together with a third zone-folded longitudinal optic (ZFLO) phonon (green line), spectral and spatial dispersion of thermal emission is further improved. Adapted with permission from ref (199). Copyright 2021 American Chemical Society.

According to the fundamental principles of statistical mechanics, any object that has a temperature greater than absolute zero (0 K) can emit electromagnetic radiation, called thermal radiation or thermal emission. Sunlight, light from incandescent bulbs, and infrared radiation from human bodies are all examples of such thermal emission. The emission patterns provided by such traditional blackbody thermal emitters are nearly isotropic, broadband, and unpolarized in the far field, which are less useful for many IR applications such as nondispersive IR sensors^205,206^ and free-space optical communications.^207^ Kirchoff’s law states that the thermal emissivity is equivalent to optical absorption at the same frequency, direction, and polarization state in a reciprocal system,^208^ and thus, the far-field thermal emission signature can be altered through engineering the optical properties of the materials. While engineered nanostructures or metasurfaces have been extensivity exploited to tailor far-field thermal emission,^209,210^ polar materials supporting SPhPs are a particularly interesting platform to control thermal radiation patterns. The seminal work by Greffet et al.^197^ demonstrated spatially coherent (directional) thermal emission from a patterned one-dimensional SiC grating with the frequency-specific emission angles diffraction induced SPhP dispersion (Figure 8a), while polarization-dependent far-field thermal emission was explored through the thermal excitation of SPhPs from a single cylindrical SiC (Figure 8b).^53^ In addition, narrowband thermal emission signatures were reported from periodic arrays of SiC bowtie nanoantennas that support localized SPhP resonances (Figure 8c).^198^ Building upon these previous works, more recently it has been demonstrated that both the angular pattern and the spectral line width of thermal emission can be further improved through the introduction of strong coupling between the propagating and localized SPhP modes together with a third zone-folded longitudinal optic (ZFLO) phonon (Figure 8d).^199^ Furthermore, it is reported that patterned phonon–polariton narrowband thermal emitters can achieve ∼1 mW/cm^2^ output power within the Restrahlen band of SiC under waste-heat operation conditions at device temperature restricted to below 85 °C.^211^ Besides polar nanostructures, far-field thermal emission from bulk low-symmetry polar crystals such as calcite also exhibits asymmetric emission patterns due to changes of vibrational modes induced by the tilted optic axis.^281^

**Figure 9:**
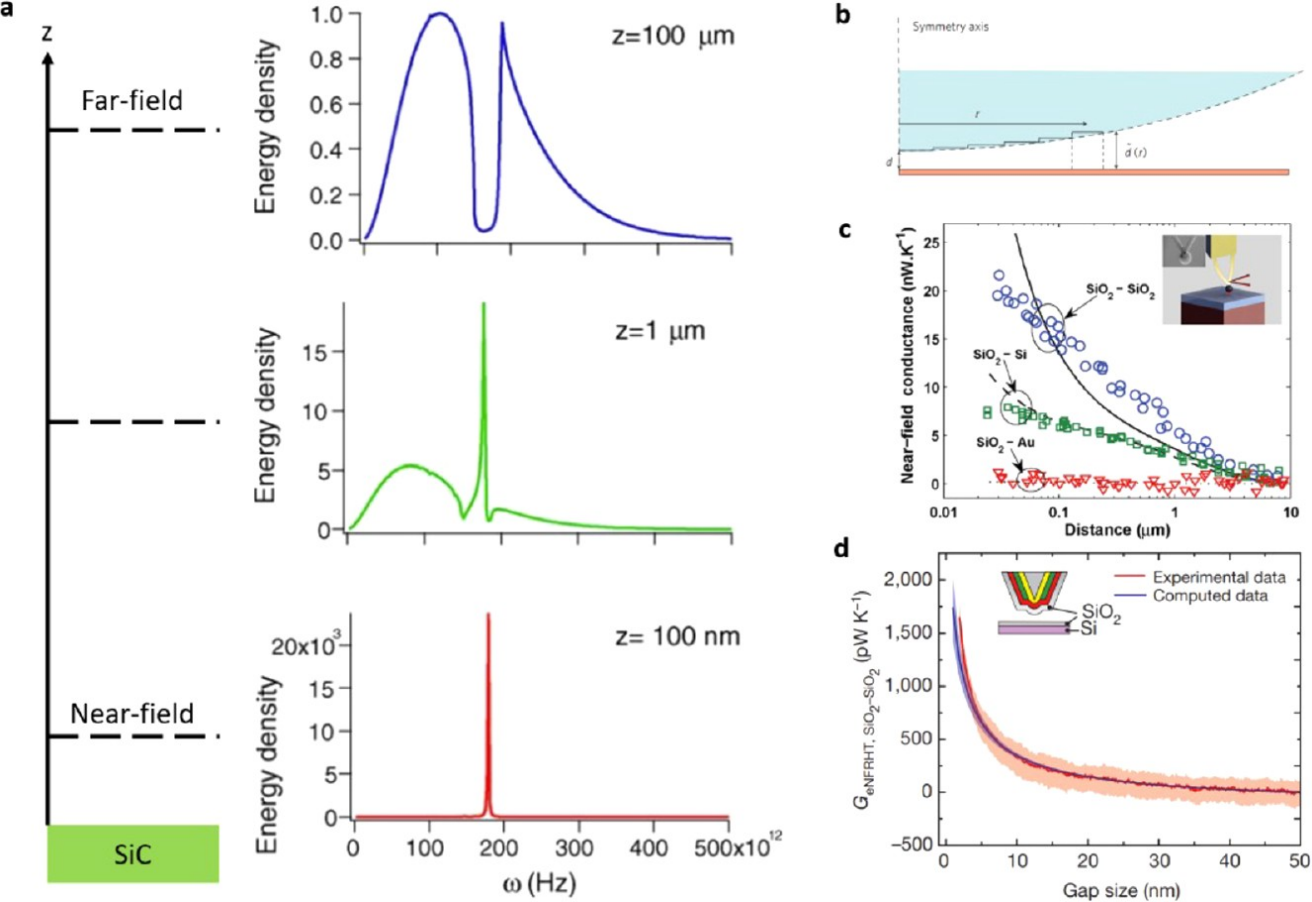
Near-field radiative heat transfer using surface phonon polaritons. (a) Calculated density of electromagnetic energy above SiC (300 K) at three different distances covering both the far-field and near-field regimes. Adapted with permission from ref (212). Copyright 2005 Elsevier. (b) A schematic of the Derjaguin approximation in NFRHT experimental verifications. Adapted with permission from ref (201). Copyright 2009 Springer Nature. (c) Experimental data from the heat transfer distance measurement and comparison with the theoretical prediction for three different material combinations. A schematic diagram of the experimental setup is shown in the inset. Adapted with permission from ref (200). Copyright 2009 American Chemical Society. (d) Measured extreme near-field radiative conductance between a SiO_2_-coated probe (310 K) and a SiO_2_ substrate at 425 K. The red shaded region and blue shaded region are the standard deviations in the measurements and calculations. A schematic diagram of the experimental setup is shown in the inset. Adapted with permission from ref (202). Copyright 2015 Springer Nature.

Far-field thermal emission follows Planck’s law, which sets an upper limit for the radiative heat transfer between objects at different temperatures. However, Planck’s law fails in a variety of situations, specifically when the radiative heat transfer process occurs between a source and sink spaced at a distance less than the thermal (so-called Wien) wavelength, which is about 10 μm at room temperature (∼300 K) according to Wien’s displacement law. Such situations are termed the near-field regime, where the fast-decaying evanescent component of the electromagnetic waves dominates the radiative heat transfer process. As SPhPs possess well-known evanescent character, i.e., evanescent surface waves, the phonon–polariton medium provides an ideal platform to explore near-field radiative heat transfer (NFRHT), which can even suppress the far-field blackbody limit from the contribution of tunneling of evanescent surface waves. To illustrate the near-field contribution in NFRHT enhancement, Greffet et al.^197^ calculated the density of electromagnetic energy above a phonon–polariton medium, SiC, at 300 K (Figure 9a).^212^ In the far-field, i.e., for a distance *z* (100 μm), the energy density spectrum resembles that of a traditional blackbody except within the Restrahlen band of SiC, where the material exhibits a negative permittivity and is therefore highly reflective (almost no thermal emission in such a spectral region). However, in the near-field, i.e., for a distance *z* smaller than the thermal wavelength (10 μm), the energy density spectrum changes dramatically, and a strong peak emerges inside the Restrahlen band of SiC. At 100 nm above the SiC surface, the thermal emission evolves to become nearly monochromatic, with the energy density increasing by more than 4 orders of magnitude at the SPhP frequency (ε = −1, where ε is the real part permittivity of SiC). Such near-field contributions from evanescent fields of SPhPs are the basis of NFRHT enhancement.

Even though NFRHT was initially theoretically predicted in the early 1970s,^213^ experimental verification of the anticipated NFRHT enhancement from SPhPs is challenging because of the necessity of achieving small uniform vacuum gaps on the order of a few nanometer separation. Initial efforts toward NFRHT were based on the Derjaguin approximation; i.e., the radiative heat transfer between a sphere and a plane is obtained by integrating the local contributions for two parallel planes with a distance of *d* (Figure 9b).^201^ Based on this approximation, the NRFHT exhibits 1/*d* dependence on sphere-plane geometry instead of 1/*d*^2^ dependence on flat plane–plane geometry. NFRHT between two phonon–polaritonic media was initially experimentally demonstrated in gaps as small as 20–30 nm,^200,201^ showing larger NFRHT enhancements when compared to other media that do not support polaritonic modes (Figure 9c). Implementing custom-fabricated scanning probes with embedded thermocouples, NFRHT for sphere-plane geometries with gaps as small as 2 nm have now been demonstrated experimentally, which exhibit much larger NFRHT enhancements (Figure 9d).^202^ Recently, NFRHT has been experimentally probed for the flat plane–plane geometry with a gap of only 30 nm, demonstrating a NFRHT enhancement on the order of 10^3^ with respect to the far-field blackbody limit.^214,215^ Beyond the planar phonon polaritonic slab approach, periodic nanostructures have also been theoretically proposed to enhance the NFRHT, with a focus on SiC-based designs.^216^

Moving beyond radiative heat transfer, conductive thermal transport is another channel for heat dissipation. Although acoustic phonons typically dominate the in-plane thermal energy transport in bulk materials, the higher phonon frequencies associated with optical phonons could offer significant implications, as these inherently offer higher heat capacities. However, optical phonons on their own suffer from near-zero group velocities near the Γ point and as such do not typically provide any substantial contributions to the thermal conductivity. On the other hand, the hybridization between such optical phonons with light in the form of SPhPs could overcome this limitation and allow these high-energy modes the opportunity to participate in the conductive heat transfer process, especially in nanostructures. In addition to the noted benefits of the high heat capacity associated with optical phonons, such SPhP-driven thermal conduction could benefit significantly from the fast (slow light, ultrafast phonon) propagation of SPhPs, with propagation lengths much larger than the typical mean free path of the optical phonon at the same frequency and the surface-confined fields associated with most SPhPs being of critical importance for this effect in nanostructures featuring a very high surface-to-volume ratio.

**Figure 10:**
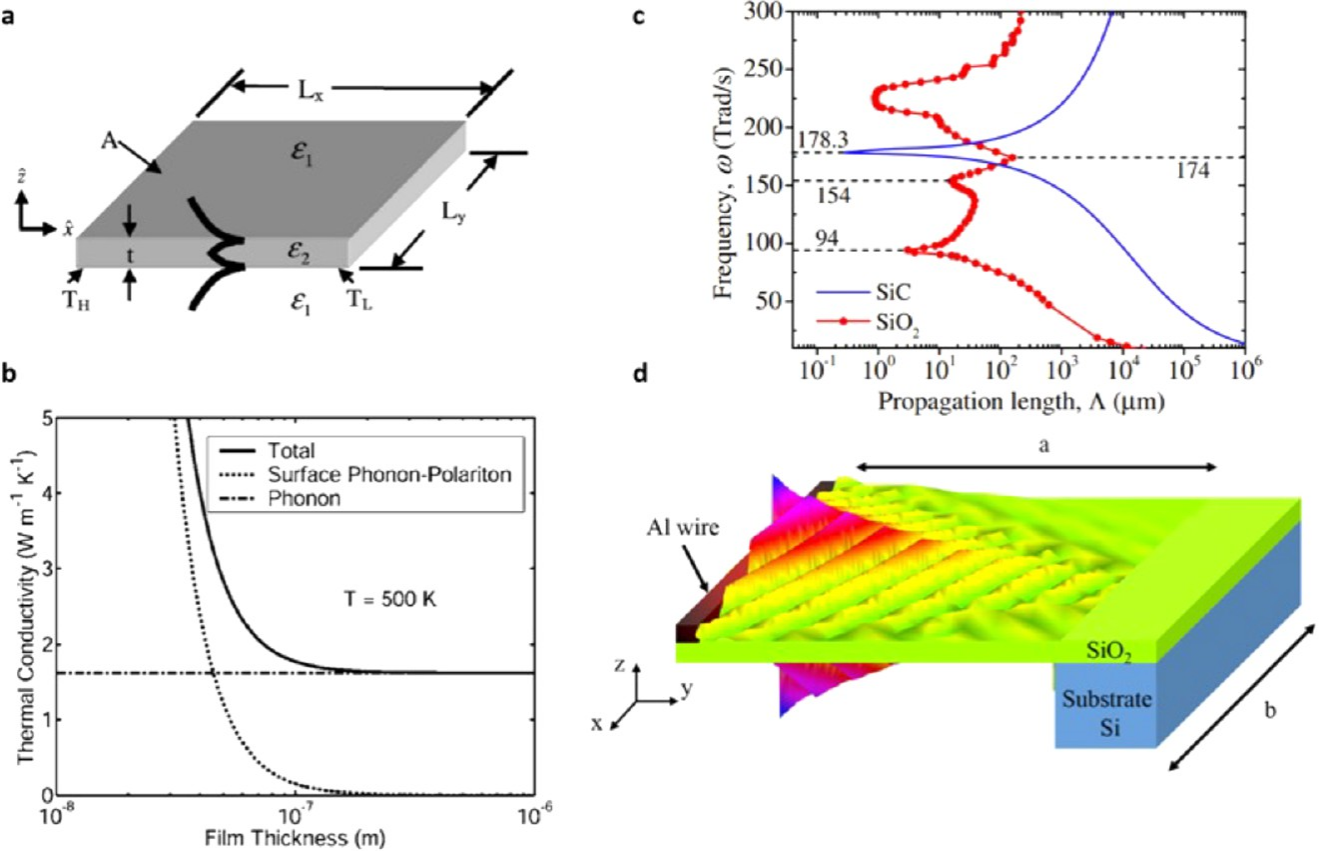
Surface phonon polariton mediated heat conduction. (a) SPhPs on both sides of a thin film of polar material can transport heat from high temperature (*T*_H_) to low temperature (*T*_L_). (b) Calculated thermal conductivity of amorphous SiO_2_ due to phonons and SPhPs as a function of the film thickness. Adapted with permission from ref (203). Copyright 2005 American Physical Society. (c) Calculated propagating length as a function of the frequency for the polar nanowires of SiC and SiO_2_ surrounded by air. Adapted with permission from ref (204). Copyright 2014 American Physical Society. (d) Schematic of the SPhPs that are thermally excited by the hot metal wire, which propagates along both sides of the membrane used in the experiments. Adapted with permission from ref (217). Copyright 2019 American Chemical Society.

It was theoretically predicted that SPhPs can contribute to the in-plane heat transfer in a thin film of amorphous polar materials, SiO_2_ in this case (Figure 10a).^203^ Employing a kinetic-theory-based approach, it was calculated that the heat flux carried by phonon polaritons (to include bulk phonon polaritons supported at frequencies below the TO phonon frequency) increases with decreasing polar film thickness and can even exceed the heat flux carried by acoustic phonon modes (Figure 10b). This seminal work brought forth an approach to boost thermal conductivity in polar nanostructures. In addition to polar thin films, the thermal conductance of one-dimensional polar nanowires with the contribution from SPhPs was also theoretically calculated.^204^ The calculated phonon polaritons propagating along polar nanowires possess ultralong propagation lengths, although some simplifications were used in the dispersion calculations (Figure 10c). Since these early theoretical predictions, initial experimental attempts have been made to explore the SPhPs mediating conductive heat transfer, for example, thermal measurements in amorphous SiO_2_ thin films (Figure 10d)^217^ and amorphous SiN nanomembranes,^218^ which were claimed to support phonon polaritons. However, due to the amorphous nature of these membranes, the permittivity does not extend to negative values, and thus, such enhancements that are observed rely entirely on the bulk phonon polaritons supported at lower frequencies than the TO phonons. While some signs of progress have been made experimentally, unambiguous experimental demonstration of the role that SPhP’s supported within the Reststrahlen band can play in dictating the thermal transport processes is still lacking. Only very recently has SPhP-mediated conductive heat transfer been reported at ultrafast time scales featuring time constants much shorter than the bulk values across gold/hBN interfaces. As this is an emerging field in the early stages, more experimental investigations together with theoretical calculations are still needed to probe SPhP-mediated heat conduction.

## V Energy Transduction among Various Phases of Matter

Thus far, this Review has focused on the energy transfer mechanisms across interfaces composed of solid density media (10^22^ atoms cm^–3^). However, as the interatomic bonds weaken and the density of a single medium comprising the interface reduces toward that of a liquid, there becomes a shift in the underlying processes that dictate energy transfer. Indeed, the mathematical framework of phonons themselves begins to breakdown; the lack of a well-defined crystalline symmetry imposes a difficulty in defining vibrational wavevectors and group velocities in amorphous solids and other low-density phases (i.e., liquids, gases, and plasmas). This is not to suggest that we simply have no theory of heat transfer in these systems. In fact, there have been a number of fairly successful frameworks that can be applied toward the modeling and understanding of thermal transport in amorphous media, such as the “model of the minimum thermal conductivity of solids”,^219,220^ in which the relaxation time of vibrational modes in a standard phonon gas model is taken to be half a period of vibrational oscillation (e.g., the carrier mean free path is limited to the distance of adjacent atoms, due to the lack of long-range order). However, there exists a nearly equivalent number of works that exemplify the inadequacies of such formalisms and approximations.^221−223^

Due to these fundamental limitations in our current theory of energy transfer within the bulk of these low-density amorphous media, one can easily imagine the increasing complications that arise in attempting to understand the intricacies of the nanoscale phenomena that dictate energy transduction across the interface from a solid to a low-density media. As such, we will focus the following section primarily on experimental measurements and advances toward understanding the energy transfer mechanisms across solid–liquid, solid–gas, and solid–plasma interfaces.

### V.a Solid–Liquid Interfaces

The understanding and manipulation of heat transfer across solid–liquid interfaces is critical toward a number of advanced applications. For example, although solid-state conduction efficiently delocalizes hot-spots in microelectronic systems, thermal accumulation within the chip quickly arises unless the heat can be dissipated over larger length scales, which is readily achieved through convective processes in liquid systems. Nonetheless, this macroscopic convective heat transfer is fundamentally limited by the rate of heat transfer that can occur from the solid device to the liquid cooling layer. Similarly, photothermal therapies rely on the rapid heating of solid micro- and nanoparticles embedded in tissues and cells that are primarily aqueous in nature; the heat flux from this nanoparticle into the surrounding tissue ultimately dictates the extent of cell-death. Thus, from the length scales of macroscale cooling of data servers to the localized heating of nanoparticles, the study of solid–liquid interfacial thermal transport is critical.

In the thermal management of most “macroscale” devices (>1 μm), there are two regimes of pertinent cooling. The first is at low-to-moderate interfacial heat fluxes, where the local liquid temperature remains well below the boiling point. The second is at increased heat fluxes, where the temperature rise is sufficient such that the liquid media undergo a phase transition to a vapor phase. In both cases, conduction from the solid to the liquid phase remains as the primary heat transfer mechanism. This should come as little surprise: the liquid-to-vapor phase transition, nucleate pool boiling, and other phenomena that increase the net heat transfer rate of the macroscopic system can only occur after sufficient energy has transferred across the solid–liquid interface itself. Indeed, such are the cases in which nucleate pool boiling arises, and the interfacial heat transfer remains fundamentally dictated by the rate at which vibrations in the solid can transfer energy to impinging gas molecules, as discussed in the following section for solid–gas interfaces. Thus, while a significant body of work has been understandably devoted toward net-heat transfer rates, ranging from clever microscale geometries and active systems^224^ down to nanoengineering of material surfaces,^62^ an understanding of the fundamental atomic-level processes that dictate solid–liquid energy transport remains imperative.

**Figure 11:**
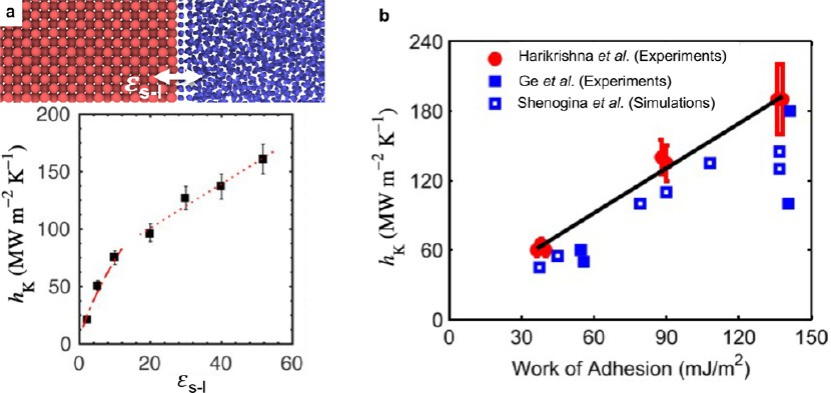
(a) Thermal boundary conductance across a Lennard-Jones-based solid–liquid interface (top panel) increases monotonically as a function of interaction strength (ε_s–l_) across the interface (bottom panel). Adapted with permission from ref (85). Copyright 2016 American Chemical Society. (b) Experimental results on the gold–water interface showing a similar monotonic increase with work of adhesion. Adapted with permission from ref (225). Copyright 2013 American Institute of Physics. The experimental results are also supported by molecular dynamics simulations by Shenogina et al.^226^

With respect to the nanoscopic mechanisms that drive interfacial energy transport, a number of recent works have pushed the theoretical understanding of vibrational scattering and energy exchange across solid/liquid interfaces.^85,227−231^ In addition to this, fundamental theory, modeling, and simulations have helped frame a basis to suggest that the strength of the bond at the solid/liquid interface, which can be determined via the equilibrium contact angle, can be correlated to the efficacy of thermal transport.^85,226,232−238^ For instance, the Kapitza conductance, *h*_K_, across a Lennard-Jones-based solid–liquid interface increases linearly with the energy parameter (ε_s–l_, representing the strength of solid–liquid interactions), as shown in Figure 11a. Similarly, recent theoretical work by Chen^239^ has extended the Boltzmann transport equation to the conditions that arise at solid–liquid interfaces, providing predictive insight toward the interfacial temperature drop based on macroscopic parameters, such as liquid density, without the need for explicit contact angle measurements.

In general, various experimental works have resulted in similar conclusions, where greater hydrophobicity at solid/water interfaces, determined via contact angle measurements, leads to a reduction in thermal boundary conductance (TBC, a measure of nanoscale energy transport across the interface).^225,226,240,241^ For instance, as shown in Figure 11b, experimental works have shown that increasing the work of adhesion via self-assembled monolayers between the gold and water interface can lead to a monotonic increase in *h*_K_ across these interfaces. In these studies, the interfacial bond strength and resultant hydrophobicity were manipulated via changes in constituents of self-assembled monolayers (SAMs) formed at the solid–liquid interface (i.e., varying the terminal group of the SAM). In general, these results imply that a smaller contact angle, and hence better “wetting”, leads to greater TBC. However, experimental works studying energy coupling across planar solid/liquid interfaces in which the liquid is not water, and relating this energy coupling to wetting, are scarce. The strengths of the intermolecular forces in a liquid relative to the interfacial bonding environment are the underlying mechanism that drives the manifestation of contact angles and wettability.^242^

This experimental scarcity can be strongly attributed to the errors and limitations associated with state-of-the-art nanoscale thermal metrology techniques, namely, laser-based thermoreflectance methods such as TDTR and FDTR. While these methods would be optimal for probing the solid–liquid interactions on the nanoscale due to its routine use in measurements of TBC mainly across solid–solid interfaces,^42^ the experimental insensitivity to the interfacial resistance posed by a solid–liquid interface, due to the large thermal resistance of liquids relative to that of the interface, make this approach prone to large uncertainties; in some cases, such as those that involve low-thermal-effusivity fluids, the TBC is simply unmeasurable with these techniques. In a recent study by Tomko et al., the experimental sensitivity and resulting uncertainty in measuring solid–liquid thermal boundary conductance via TDTR measurements was surveyed.^243^ The relative sensitivity of the interfacial resistance is significantly lower compared to that of the liquid thermal conductivity for both water (κ = 0.6 W m^–1^ K^–1^) and the common refrigerant, a fluorocarbon-based liquid, FC70 (κ < 0.1 W m^–1^ K^–1^); these results suggest that only a lower bound of TBC can be recovered in such solid/liquid systems with TDTR. Rather, the authors provide alternative laser-based methods, such as picosecond acoustics^244,245^ and laser ablation,^246^ as a means of extracting the relative TBC across different solid/liquid interfaces. However, in the case of extremely high interfacial resistances, these methods are certainly applicable. For example, Yu et al.^247^ found through the introduction of nonane, a hydrocarbon ideally representative of molecules that would arise in oil immersion environments, an Au/water TBC of 610 kW m^–2^ K^–1^—a resistance certainly large enough to overcome typical experimental uncertainties.

Even with experimental uncertainties in planar geometries, these thermoreflectance methods remain well-suited for the measurement of solid–liquid TBCs in colloidal nanoparticle systems. Indeed, in the limit of which the nanoparticle radius is such that it can be considered a point source with respect to the surrounding fluid, the thermal model for extracting TBC is simplified with respect to the analysis required for thin films that undergo bidirectional heat transfer into both the supporting substrate and the overlying liquid of interest. Rather than requiring a multilayer thermal model, with numerous thermophysical inputs associated with each layer, for colloidal systems with sufficiently small particle diameters, the thermal relaxation following pulsed excitation can be modeled as an exponential decay in time.^248^ In this limit, the thermal boundary conductance at the nanoparticle/liquid interface is given by , where *r* is the particle radius, *C*_*v*_ is the particle’s volumetric heat capacity, and τ is the thermal decay time constant. This method has been widely applied to a number of colloidal particle systems, providing, arguably, the largest breadth of experiments associated with nanoscale solid–liquid interfacial thermal transport; these studies include metal nanoparticles composed of various elements,^248^ nanorods and nanotubes,^249^ and suspended molecules.^250^ The primary limitation of this approach is the viability of suspending such particles in the fluid of interest. For example, it is difficult to manufacture colloidal metal nanoparticles lacking an adhesion layer in an arbitrary liquid of choice, thus making this approach inapplicable for the study of a “bare” metal/liquid interface.

It should be noted that the majority of the preceding discussion and works have focused on vibration-mediated heat transfer processes due to the fact that they carry a large majority of the associated energy flux. However, this is not to suggest that electronic and photonic energy transport does not arise across such interfaces; this should come as no surprise due to the fact that application spaces such as electrochemical and photochemical catalysis both rely on interfacial charge transport and sustain large optical-mode activity for radiative transport. Indeed, a recent review by Lee et al.^251^ on the role of interfacial hot-electron processes across solid–liquid interfaces provides an in-depth survey of many recent works. Nonetheless, the heat flux of such processes remains quite small relative to vibrational energy transport based on our current understanding; further interfacial engineering and excitation may be likely to increase the contribution of electron-mediated heat fluxes across solid–liquid interfaces and thus deserves continued exploration. Additionally, radiative processes, particularly those under near-field conditions that are known to enhance solid–solid interfacial energy transfer as reviewed in Section IV, have been relatively unexplored for solid–liquid interfaces. This is likely due to two reasons: (i) experimental methods for investigating surface phonon modes in solids, such as the scanning near-field optical microscopy,^252^ are ill-equipped for liquid-phase studies, and (ii) the potentially weak collective oscillations that exist in liquid phases. Nonetheless, recent work by Wang et al.^253^ has developed a liquid-phase peak force infrared (LiPFIR) method capable of performing near-field measurements within liquids, providing *in situ* measurements of h-BN PhPs, thus displaying accessibility toward overcoming the experimental hurdle. Further, many liquids in theory should sustain phonon polaritons based solely on the fact that they are polar; the lack of crystalline order is by no means a requisite for polariton excitation, as exhibited even through enhanced heat transfer rates in amorphous materials nearly two decades ago.^254^ Indeed, detailed analysis on the optical activity paired with molecular dynamic simulations performed by Elton and Fernández-Serra^255^ has shown that the network of hydrogen bonds in liquid water leads to dispersive optical modes, thus exhibiting (transverse-longitudinal) optical mode splitting and vibrational propagation. The aforementioned advances in metrology methods capable of liquid-phase near-field measurements paired with solid–liquid interfacial engineering and microfluidics should provide a test-bed for further exploration of true liquid-sustained SPhPs, potentially providing a means of overcoming the intrinsically low thermal conductivity of many liquids.

### V.b Solid–Gas Interfaces

The exchange of energy between a solid surface and adjacent gas molecules is a prerequisite for a range of fundamental processes and applications, including thermal mitigation of electronic devices and nanostructures via convection, deposition methods accessed with vapor adsorption (both chemical and physical techniques), and even for consideration of drag coefficients in moving bodies.^256^ At a continuum level, this interfacial heat transfer is understood through convection, as the coupled heat-mass transfer of a moving fluid is the dominant heat transfer process; this is similar to our case of solids, where the interfacial thermal resistance plays a negligible role until the dimensions of the system are greatly reduced. Indeed, non-continuum heat transfer effects arise when the characteristic length scale of the system becomes comparable to the mean free path of the gas. Thus, a detailed description of the nanoscopic energy transfer mechanisms across a solid–gas interface is necessary in not only systems with reduced dimensionalities (e.g., nanostructures) but also systems immersed in low-pressure gases due to the inverse relationship of gas pressure and the molecular mean free path. To gain insight into the rate of energy transfer across a solid–gas interface, we can consider a flux of gas molecules impinging upon a solid surface. If the incident gas molecules were to “stick” to the surface without any reflections, the entirety of the molecule’s momentum, and thus energy, would have been transferred to the solid body. In this simple scenario, the rate of energy transfer is nothing more than the incident energy flux (e.g., the average energy per gas molecule multiplied by the particle flux). In a more physically relevant scenario, where the molecules only transfer a fraction of their energy and reflect upon interaction with a solid surface, it should not be surprising that the primary descriptor for this non-continuum rate of energy transfer at a solid–gas interface is given by , where *E*_i_ and *E*_f_ are the average molecular energies of the incident and scattered gas particles, respectively, and *E*_s_ is the energy flux that would be achieved if the reflected molecules were in thermal equilibrium with the solid surface post-scattering. The descriptor, α, varies from zero (adiabatic, specular reflection) and unity (perfect accommodation) and is termed the accommodation coefficient. Not only is this formalism reliant upon the flux of gas molecules being well-described by a single temperature (or average energy), but it should be noted that it typically does not provide *a priori* prediction of heat fluxes unless a pre-existing, detailed knowledge of solid–gas interactions is already known.

It is important to note the extensive volume of literature that has investigated this specific exchange of kinetic energy at the solid–gas interface via beam scattering experiments.^257−260^ These ultrahigh vacuum studies are typically performed on a “clean” metal surface, where the greatly reduced pressures facilitate ballistic propagation of gas molecules from a source to the sample as well as the sample to the detector. These studies have provided valuable insight into gas–surface interactions; for example, these studies have demonstrated that kinetic energy is more readily transferred when the mass of the incident atom is similar to the mass of the atoms comprising the solid surface.^261,262^ Despite such insight, it is critical that a similar understanding of gas–solid dynamics is achieved at near-atmospheric pressures, due to both technological relevance and the transfer to a diffusive nature of the interactions. To this end, there is an intrinsic difficulty in experimentally resolving the energy transfer rates at solid–gas interfaces under ambient conditions. First, the measurement must have spatial resolution on the order of the characteristic length scale of energy transport (∼submicron for atmospheric conditions); the experimental method must be capable of resolving both flux and temperature distributions at this scale without altering heat flow. Similarly, the temporal resolution must be adequate for resolving the proper mechanisms of heat transfer at the solid–gas interface; at ultrafast (e.g., TDTR) time scales, there is insufficient time for mass transport to occur, and the technique is only capable of resolving conductive heat transfer mechanisms. On the contrary, steady-state methods can measure a heat transfer coefficient that is dominated by convection, as it is the primary heat transfer pathway in gases due to the phase’s intrinsically low thermal conductivity. Lastly, due to the comparatively low heat transfer coefficients associated with solid–gas interfaces (<1 MW m^–2^ K^–1^) relative to those at solid–solid interfaces (typically >25 MW m^–2^ K^–1^), or conduction in bulk solids, it is difficult to decouple the weak heat loss to the gas relative to that of the supporting substrate, as similarly found in the case of solids.

Atomistic modeling of solid–gas TBC has also provided important physical insights into the heat transfer mechanisms at these interfaces.^263−268^ For example, Liang et al.^263^ investigated the effect of interfacial parameters on the thermal accommodation coefficient (TAC) at the solid–gas interface, which revealed that the TAC on a smooth and perfect interface is significantly lower than that on a disordered interface. This was attributed to the roughness and defects on the disordered interface, which are more conducive to diffuse scattering of gas molecules. However, these physical mechanisms can be drastically different when the gas molecules are confined in the nanopores of a solid framework. In this regard, Feng et al.^268^ conducted non-equilibrium molecular dynamics simulations on polymer surfaces exposed to various types of gases and found that, when the gases are confined to 10 nm pores, the gas thermal conductivities can be as much as 3 orders of magnitude lower than their bulk values, providing important design criteria for thermal insulation materials.

In porous materials, heat transfer is mainly contributed by conduction through the solid framework and the gas particles, and also partially via radiation through the voids.^269^ Generally, there is a trade-off between reducing the solid thermal conductivity by increasing porosity, but it also leads to enhanced contributions from the gas and radiative heat transfer channels. More recently, the control of the relative contributions of the solid framework and the infiltrated gases to the overall heat transfer has been demonstrated in two-dimensional (2D) covalent organic frameworks (COFs).^270,271^ Specifically, it has been shown that the solid–gas interactions in the one-dimensional pore volumes of the COFs can lead to additional conductive heat transfer channels associated with the gas molecules that facilitate heat transfer along the pores.^270,271^ Through increased collisions with the solid framework of the COFs, confined gas molecules have been shown to conduct heat along the 1D pores (that resemble “chimneys”) of COFs to enhance their thermal conductivity. However, if the pores are smaller than or comparable to ∼1.5 nm, the solid–gas interactions are shown to lead to reduction in the overall thermal conductivity via solid–gas scattering.^270^ These results highlight the prowess of molecular dynamics simulations in providing unparalleled atomistic insights into heat transfer mechanisms for various solid/gas systems and can have major implications for advancing some very important next-generation technologies such as those based on 2D COFs for catalysis and gas storage applications.

### V.c Solid–Plasma Interfaces

With regard to the mechanisms that drive interfacial energy transport, plasmas lie at an intriguing intersection between that of metallic solids and that of a free gas. Plasma is an ionized gas that contains approximately equal numbers of positively and negatively charged species. Typically, these charged particles are in the form of positive ions and free electrons, which are created by adding energy, either thermal, light, or electric, to a gas volume to ionize the gas. A distinction that separates this fourth state of matter from a gas is that, while the plasma volume is electrically neutral, there are enough charged particles to make the plasma conductive. The plasma state is the most common form of matter in the universe, with stars and the accompanying interstellar medium comprising most of this matter. In contrast, plasmas rarely occur on earth; lightning and auroras are examples. Accordingly, the number density and energy of the ions and electrons, neutral gas density and temperature, and other properties of a plasma can vary widely.

The properties of plasmas, however, make them a powerful tool in advancing technological applications that range from energy to the manufacture of commonplace materials and devices. Indeed, man-made plasmas have found uses in, for example, fusion energy, lighting, and wide screen televisions. Plasma-based material processing, on the other hand, is employed to synthesize and modify materials used for the production of hard, barrier, and multifunctional coatings, optical elements, and sensing platforms. Perhaps the most well-recognized application for plasma processing is in the semiconductor industry, where plasma processing is an indispensable tool in the fabrication of computer chips.

**Figure 12:**
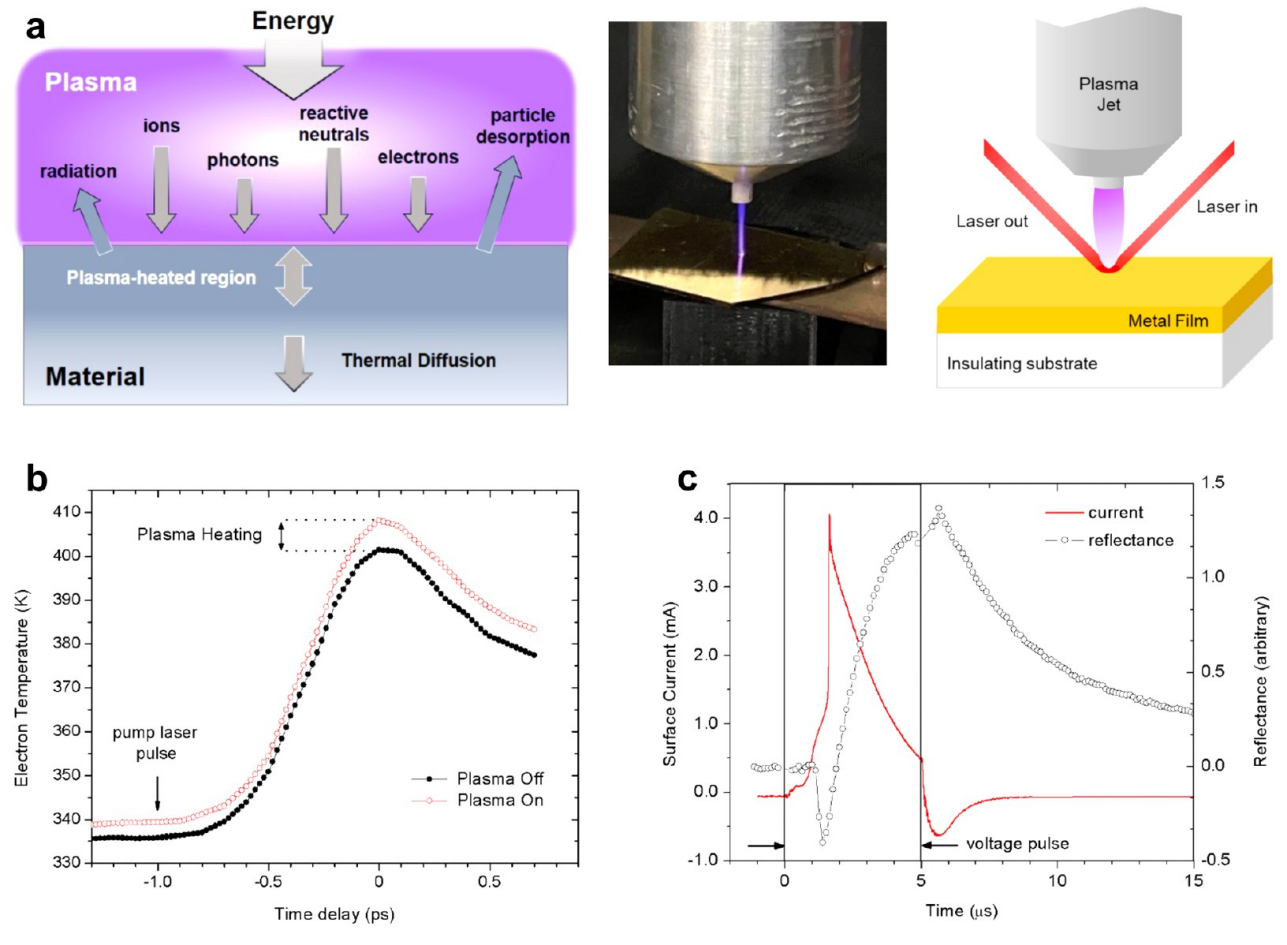
(a) A schematic representation of the plasma surface interaction, including the primary energy carriers responsible for heating and cooling the surface. Also shown is an image of the plasma jet used in refs (57) and (58), along with a schematic of the thermoreflectance measurements from ref (58). Adapted under the terms of the Creative Commons CC BY license from ref (58). Published 2022 Springer Nature (b) The TDTR results from ref (57) showing the increase in electron temperature within the material as a result of plasma exposure. Adapted with permission from ref (57). Copyright 2018 American Institute of Physics. (c) The time-depdendent thermoreflectance and the measured surface current produced by a flux of ions delivered by the pulsed plasma jet in ref (58). Note that surface heating follows the rapid rise in ion flux, while the prominent dip in reflectance preceding the increase in reflectance indicates surface cooling. From ref (58), surface cooling is associated with photons that arrive prior to the charge particle flux. Adapted under the terms of the Creative Commons CC BY license from ref (58). Published 2022 Springer Nature.

The most common types of plasmas used in materials processing are non-equilibrium, low-temperature plasmas, which are typically produced by applying an electric field to a gas volume. The field accelerates the electrons to an energy sufficient to ionize a fraction of the gas and, thus, sustain the plasma. The resulting electron energy is much greater than the heavy ions, which like the neutral background gas are at or near room temperature. When produced in a molecular gas background, the energetic electrons will also excite and dissociate a fraction of the gas molecules. As such, these plasmas can deliver a rich flux of species to a material surface including positive ions, negative ions, and electrons; charged and neutral reactive radicals; excited ions and neutrals; and a flux of photons created when excited species decay. These are depicted in the schematic of Figure 12a, which shows the various plasma species and related physical processes to consider when plasmas interact with surfaces.

In order to preserve the neutrality of the plasma, an electrical potential forms at the surfaces exposed to plasmas, which serves to accelerate the positive ions toward the surface (and decelerate the electrons) and thus equalize the flux of positive and negative charges leaving the plasma. This potential scales with electron energy but will typically cause ions to impact the walls with kinetic energies above their thermal values. The synergistic effects associated with the simultaneous delivery of both chemically active species and energy to a material’s surface is an attribute that separates plasma-based approaches from other processing techniques.^272^ Energy delivery is an essential component in driving physical and chemical processes either directly through particle and photon interactions or indirectly via surface heating. In particular, the energy flux serves to drive the surface out of thermal equilibrium with the bulk material, thus enabling local physicochemical processes that can be exploited to remove (etch) substrate material, add (deposit) different materials, or chemically modify the surface. This is distinct from heating a material to enhance surface reactions, where the entire material and/or environment is elevated in temperature. It is also a cumulative result of numerous energy transfer events associated with the individual particles and photons. As such, there is a need to develop approaches that provide a direct measure of the localized and transient energy transport mechanisms associated with the flux of species at the solid surface.

A number of approaches have been aimed at understanding and quantifying the energy flux and associated energy transfer mechanisms at the solid–plasma interface, for both low-pressure^273,274^ and atmospheric-pressure^275^ plasma systems. One can begin to understand the response of a material immersed in a plasma by considering the power balance at the surface,^273^ given by *P*_in_ = *P*_heat_ + *P*_out_. Here *P*_in_ is related to the energy flux (or power flux density) into the material, *P*_out_ is related to the energy flux leaving the material, and *P*_heat_ is the power that heats the material. *P*_in_ is determined by accounting for all collisional and radiative sources of energy from the mix of charged particles (electrons and ions), reactive gas molecules and atoms, and excited species generated in the gas phase that will either impact the material surface or produce photons that reach the surface. Also included are exothermic chemical and physical reactions at the materials’ surface and system controls such as substrate heating and/or biasing. Similarly, *P*_out_ is determined by accounting for all the processes that remove energy from the surface including particles that leave the surface (or diffuse into the bulk), radiation, endothermic reactions, and system components that serve to cool the material. While it is a difficult task to properly account for all sources that contribute to *P*_in_ and *P*_out_ in even the simplest plasma processing environments, their difference (*P*_heat_) is accessible by monitoring the temperature of the materials exposed to the plasma using . Here, *m*_*s*_ is the mass, *C*_*s*_ is the specific heat capacity, and *T*_*s*_ is the temperature of the material exposed to the plasma. While of practical interest, it is important to note that temperature is a macroscopic property that describes the statistical distribution of particle energies within the material. It is not a direct measure of the energy flux or transfer but rather the result of the energy transfer from the plasma to the surface. To address this, devices and techniques have been developed^276−278^ to measure the thermal flux directly by measuring the change in temperature over some distance or time.

Despite the advances in technology, measuring the plasma’s energy flux to a surface remains challenging. To understand this, consider that most particle energy is delivered and adsorbed rapidly within a few nanometers of the surface. For example, Graves and Humbird^272^ estimate that Ar^+^ ions with 200 eV of kinetic energy release most of their energy within about 25 Å of the surface in about 10^–12^ s. Anders^279^ argues that the potential (ionization) energy is released at (or very near) the point of contact during neutralization of ions and absorbed locally within the same time scale. Similar scaling can be expected for other particles possessing kinetic energy (e.g., fast neutrals) or potential energy (e.g., metastables). Photons will typically deliver their energy much deeper than low-energy particles, with a penetration depth that depends on wavelength. However, when they do lose their energy in the material, it is a nearly instantaneously transferred. Energy transport in a solid is largely mediated by electrons and lattice vibrations or phonons, which are typically characterized by mean free paths on the order of several to hundreds of nanometers and relaxation times that range from several femtoseconds for electrons to picoseconds or even nanoseconds for phonons.^280^ In other words, the time required to propagate energy away from a plasma-interaction zone near the surface, determined by the electron and phonon diffusion times, can be much longer than the typical times associated with *P*_in_. Thus, the energy delivered by the plasma produces a well-localized “thermal spike” near the surface that exceeds the average temperature of the material and will then dissipate in time and space. The spatiotemporal characteristics of this thermal spike and associated kinetics are not easily accessible with conventional temperature measurements using thermocouples or radiation. As such, an increasing body of work has begun investigating the surface heating and resulting energy transport processes of the thermal energy carriers in materials exposed to plasmas.

A study by Walton et al.^57^ applied a femtosecond pump–probe technique, TDTR, to measure the electronic response of a gold film exposed to an atmospheric pressure plasma jet (an image of the jet can be seen in Figure 12a). This study provided the initial, to the best of our knowledge, measurement of the response of the fundamental carriers (e.g., electrons and phonons) during plasma–solid interactions. They found that the thermoreflectance signal of the gold film (as shown in Figure 12b) is generally greater with the plasma jet incident on the sample. They also showed that the signal was maximized when the laser and jet points of contact were spatially coincident. This indicated that the exposure to the plasma jet resulted in a higher electron temperature in the material. However, the TDTR approach relied on time-averaged lock-in amplification and thus only provided insight into the time-averaged material response. While these studies are certainly of value, a direct measurement of the material response associated with the changing flux of species at the surface is required to separate the localized and transient energy transport mechanisms from the spatially and temporally averaged power transfer and temperature rise. In a follow-on work, Tomko et al.^58^ developed a method to directly interrogate the metal response with nanosecond resolution, thus providing a direct measurement of the electron temperature in the material where the jet intersected the material (a schematic of the thermoreflectance measurement is shown in Figure 12a). They associated this with the time-varying flux of carriers impinging on the solid (Figure 12c). Their time-resolved thermoreflectance method, reliant on periodic waveform analysis, found excellent agreement between measured surface current and temperature rise; the authors found that charged carriers were the primary method of transferring energy to the surface, as suggested by many earlier steady-state studies. However, with temporal resolution, the authors found a time frame during which the material surface was transiently cooled through plasma exposure; this effect had been “averaged-out” in earlier studies and is likely the result of photodesorption of adsorbed species due to photons created during the first tens of nanoseconds following plasma ignition.

Berrospe-Rodriguez et al.^59^ recently used Raman thermometry to measure the thermal response of multilayer graphene on a copper substrate during plasma exposure. Like the thermoreflectance techniques employed by Walton et al.^57^ and Tomko et al.,^58^ the approach offers a noncontact spatially resolved technique to interrogate the temperature of a material. The use of graphene multilayers, with a strong and well-characterized Raman signature, provides a means to isolate the temperature response of the surface (vs the bulk metal). The results suggest a substantial increase in the graphene temperature above the underlying substrate, which varies with plasma operating conditions. Importantly, the increase in the temperature with increasing applied power indicates a correlation between the fluence of energetic and reactive species and surface temperature.

While these studies are limited in their scope, the promise of noncontact methods such as the thermoreflectance technique is substantial. Indeed, the ability to characterize surface heating over physical and temporal scales associated with the flux of energy carriers delivered by the plasma, as well as the ability to monitor the response of electrons and phonons in the material, should provide valuable insight into the physical and chemical processes that arise during plasma exposure.

## VI Summary

We reviewed and provided our perspective on the topic of ultrafast energy transduction mechanisms across various types of material interfaces. Specifically, we focused our discussions on the role of coupled energy states dictating interfacial energy transport. These include interfacial thermal transport mediated through hybridized and localized interfacial phonons, electron–phonon coupling at solid/solid interfaces, surface phonon polariton-driven heat transfer, and thermal transport across different phases of matter including energy transfer processes at the solid–plasma interactions. For purely phonon-driven thermal boundary conductance, interfacial vibrational modes that do not exist on either “bulk” material have been shown to contribute substantially to interfacial heat flow. These emergent localized modes can dictate the vibrational physics and even the overall heat conduction in short-period superlattices. For example, the emergence of hybridized phonon modes in superlattices with high interface densities leads to rich phonon physics such as the demonstration of Anderson localization of phonons in aperiodic superlattices and the crossover from the incoherent to the coherent regime of phonon transport in superlattices with systematically varying period thicknesses. Along with the significant role of interfacial modes in dictating heat transfer across solid interfaces, hybridized phonon modes across interfaces have also been leveraged to tune the thermal conductivity of nanophononic metamaterials and appear to be a robust strategy to manipulate the entire phonon spectrum in these materials.

Studies have also shown the prospect of energy transduction from hot electrons coupling across the interface with the phonons of the material on the other side. This process is highlighted by the experimental observation of ballistic thermal injection at metal/doped non-metal interfaces, which was used to create long-lived hot electrons in the non-metal to ultimately control the plasmonic absorption and offer an approach to thermally modulate plasmon resonances in the non-metals. Simultaneously, the development of *ab initio* quantum dynamics simulations has also tremendously increased our understanding of coupled electron–phonon processes across material interfaces. For example, these first-principles-based calculations have shed light on the direct plasmon-induced charge transfer mechanisms across metal/semiconductor interfaces, a process that had been theorized previously but never rigorously studied with first-principles. As charge–charge scattering is much faster than the energy transport by phonon–phonon scattering, a sufficiently high concentration of charge carriers in the semiconductor is required to transfer a significant amount of energy across the metal/semiconductor interface. It is also possible that the hot electrons generated in a semiconductor can transfer charge or energy into a metal where participation of plasmon-like acceptor states in the metal aids in the overall energy transport process.

Surface phonon polaritons, where photons are coupled to optical phonons, are also rapidly gaining more attention for the purposes of thermal radiation and thermal conduction at surfaces and interfaces. For instance, near-field radiative heat transfer originating from the contribution of tunneling of evanescent surface waves and surface phonon polaritons in periodic nanostructures has been proposed to enhance energy transport with a strong focus on SiC-based designs. Surface phonon polariton mediated conductive heat transfer has been demonstrated as a viable route that occurs through the hybridization of light with optical phonons that possess higher heat capacities. However, these optical phonon modes inherently have slower group velocities as compared to the dispersionless acoustic modes and, therefore, typically do not substantially contribute to heat conduction in typical crystalline semiconductors. Thus, overcoming this limitation of the intrinsically localized nature of the majority of optical modes and allowing these high-energy modes the opportunity to participate in the conductive heat transfer processes can lead to dramatic enhancements in surface phonon polariton-driven heat transfer. In addition to the noted benefits of the high heat capacities associated with optical phonons, such surface phonon polariton-driven thermal conduction could benefit significantly from the fast (slow light, ultrafast phonon) propagation of these coupled energy carriers with propagation lengths much larger than the typical mean free path of the optical phonons at the same frequency. This will be of critical importance for heat conduction in nanostructures featuring a very high surface-to-volume ratio.

We have also reviewed the energy transfer mechanisms across interfaces composed of various phases of matter. Although thermal conductance across solid/liquid and solid/gas interfaces has been shown to be substantially lower than that across solid/solid interfaces, physical phenomena such as thermal accommodation in solid/gas interfaces and the ability to tune thermal conductance across a wide range through solid–liquid interactions have led to a considerable amount of research for these interfaces. For example, in nanoporous materials such as covalent organic frameworks, heat transfer efficacy can be substantially increased by inducing strong solid–gas interactions along the laminar pores; the gas adsorbates present additional heat transfer channels, where these gas molecules can conduct heat via frequent and strong collisions with the pore walls. Likewise, the flux of energy carriers delivered to the surface of plasma-exposed materials has also recently been shown to drive the physical processes at the solid/plasma interface. For instance, the interaction of a pulsed plasma jet with a metallic solid surface has been shown to include periods dominated by both heating and cooling processes. Taken together, these intertwined mechanisms of coupling between different energy carriers at interfaces and surfaces in nanomaterials are the foundation of modern technologies that are continuing to provide transformation benefits in a plethora of applications, and as such, further advancement in research in all of the aforementioned topics is a prerequisite to realize the potential of manipulating these processes for enhancing our current technology.

## Vocabulary

*Coupled local equilibria (CLE)*: When two populations of carriers are both well described by only slight perturbations from their respective equilibrium distributions, yet each of their equilibrium distributions is defined by statistically and significantly different temperatures relative to each other.

*Interfacial hybrid phonon modes*: At the interface or atomic junction between two materials, the non-intrinsic and heterogeneous masses and force constants do not intrinsically exist in the “bulk” of either homogeneous material. When degenerate with Bloch phonon waves in even one of the materials adjacent to the interface, these hybrid and interfacially localized phonon modes are resonances that show localized behavior near the interface but are smoothly connected to propagating phonon states in the material.

*Ab initio quantum dynamics simulations*: This simulation approach provides a foundational perspective on the evolution of hot carriers coupled to vibrational motions by creating a time-domain atomistic description, most closely mimicking time-resolved experiments. This computational approach combines real-time time-dependent DFT for the evolution of the electrons with non-adiabatic molecular dynamics for the evolution of ionic cores and electron– vibrational interactions.

*Ballistic thermal injection (BTI)*: A process for energy transfer across metal/semiconductor interfaces during conditions of CLE. This process begins with hot-electron generation in the metal, and prior to the electron–phonon coupling (less than a couple of picoseconds), energy propagates ballistically toward the metal/semiconductor interface. The electron energy front reaches the interface, whereby the electrons transfer their energy, rather than charge, to the pre-existing free electrons in the semiconductor’s conduction band, thus relying on electron–electron thermal boundary conductance at the metal/doped semiconductor interface. The pre-existing semiconductor’s electrons are now at an elevated temperature.

*Phonon polaritons*: Quasiparticles comprised of strongly coupled photons and optical phonons.

*Reststrahlen band*: Narrow energy band in a given medium where the real part of the permittivity tensor becomes negative, resulting in the inability for electromagnetic radiation to propagate.
